# Green Tea and Decaffeinated Light Roasted Green Coffee Extract Combination Improved Cardiac Insulin Resistance through Free Fatty Acids and Adiponectin/FAS Pathways Amelioration in Metabolic Syndrome Rat Model

**DOI:** 10.12688/f1000research.55470.2

**Published:** 2023-09-14

**Authors:** Mifetika Lukitasari, Mohammad Saifur Rohman, Dwi Adi Nugroho, Mukhamad Nur Kholis, Nila Aisyah Wahyuni, Nashi Widodo

**Affiliations:** 1 Department of Nursing, Faculty of Medicine, Brawijaya University, Malang, East java, +62, Indonesia; 2 Department of Cardiology and Vascular Medicine, Faculty of Medicine, Brawijaya University, Malang, East java, +62, Indonesia; 3 Department of Herbal Medicine, Brawijaya Cardiovascular Research Group, Faculty of Medicine, Brawijaya University, Malang, East java, +62, Indonesia; 4 Magister of Biomedical Sciences, Faculty of Medicine, Brawijaya University, Malang, East java, +62, Indonesia; 5 Department of Biology Mathematics and Natural Sciences, Brawijaya, Malang, East java, +62, Indonesia

**Keywords:** metabolic syndrome; green tea; green coffee; cardiac insulin resistance

## Abstract

**Background**: Insulin resistance has been independently associated with cardiac diseases. A free fatty acid is recently known to induce cardiac insulin resistance due to low-grade inflammation. Therefore, the improvement of free fatty acid levels can also improve cardiac insulin resistance. This study investigated the combination of green tea and decaffeinated-light roasted green coffee extract in improvement of free fatty acid-induced cardiac insulin resistance by improving the adiponectin/FAS pathway.

**Methods:** This study used 25 males Sprague-Dawley rats induced by a high-fat high sucrose diet and injection of low dose streptozotocin to make a metabolic syndrome (MS) rat model and standard chow as healthy control rats. The MS rats were treated with green tea (200 mg/ b. w.), decaffeinated-light roasted green coffee (300 mg/ b. w.), and the combination of both extracts in 9 weeks. Experimental groups in this study were divided into 5 groups: 1) MS (HFHS diet + STZ) group, 2) NC (normal chow) group, 3) GT (green tea extract) group, 4) GC (decaffeinated-light roasted green coffee extract), 5) CM (combination of both extracts) group. Adiponectin and HOMA-IR level was analysed using ELISA, and the gene expression of *Adipo-R1, FAS, PI3K, PDK1, Akt, GLUT4* was measured by RT-PCR.

**Results:** The combination of green tea and decaffeinated-light roasted green coffee showed synergistic effects in improving FFA levels. The adiponectin/FAS pathway was attenuated in the CM group. Moreover, the combination also showed improvement in cardiac insulin resistance markers such as IRS1/2, PI3K, PDK1, Akt, and GLUT4.

**Conclusions**: The combination of green tea and decaffeinated-light roasted green coffee extract improved cardiac insulin resistance better than green tea and green coffee extract administration alone by reducing free fatty acids levels through adiponectin/FAS pathway modulation.

## Editorial note

Editorial Note (4 ^th^ August 2023): The F1000 Editorial Team has not yet received a new version of this article, as detailed in the Editorial Note published on 16 ^th^ June 2023. The F1000 Editorial Team is actively contacting the authors to request the new version of the article. Peer review activity remains suspended until the authors publish a new version of this article.

Editorial Note (16 ^th^ June 2023): Since publication, it has been brought to the attention of the Editorial Team that the article was missing key information regarding animal treatment and ethical approval. The Editorial Team requested further detail and an explanation from the authors in March 2023. The authors provided an adequate response and were requested by the Editorial Team to create a new version of the article to include the additional details. Peer review activity has been suspended until the authors publish a new version of this article.

## Introduction

The incidence of metabolic syndrome (MS) features, such as obesity, hyperglycemia, and insulin resistance has increased and is associated with excessive food consumption. ^1^ MS prevalence has increased about 50–75% in the last decade. ^2^ A previous study showed that insulin resistance had been known to be a significant predictor of heart failure (HF) ^3^ and the major feature in HF patients. ^4^ Many studies have shown that insulin resistance might develop cardiac remodellings such as hypertrophy, fibrosis, and cardiac dysfunction without coronary artery disease or hypertension. ^5^^–^^9^ Lipotoxicity by free fatty acids (FFAs) has been the primary cause of insulin resistance ^10^ through low-grade inflammation in cardiac tissue. ^11^ Kim *et al.* (2009) ^12^ reported that FFAs might cause inflammation and suppress glucose uptake through inhibition of IRS-1 in cardiac tissue. Meanwhile, reducing FFAs plasma levels could improve cardiac function in the high-fat diet-induced obese rat model. ^13^ Therefore, improving the lipid profile could prevent the development of cardiac insulin resistance.

Recently, natural compound derivatives revealed beneficial effects in metabolic syndrome improvement. One of the natural compounds that are widely consumed is epigallocatechin-3-gallate (EGCG) which is mainly derived from green tea leaves. ^14^ Many studies have investigated the beneficial effect of green tea administration and its constituents in alleviating metabolic syndrome. Most of those studies revealed that green tea administration modulated insulin sensitivity, reduced blood glucose level, and improved lipid profile in metabolic syndrome animal models. ^15^^–^^17^ Additionally, FFAs-induced insulin resistance and hyperglycemia-induced cardiac fibrosis could be attenuated by EGCG administration *in vivo*. ^18^ Moreover, chlorogenic acid (CGA), a phenolic compound in green coffee beans, has been another natural compound derivative that could alleviate metabolic syndrome. ^19^^,^^20^ CGA potentiates insulin activity similar to the therapeutic mechanism of metformin ^21^ but does not induce obesity, unlike thiazolidinedione (TZD) or insulin therapy. ^22^ A previous study were also showed that CGA improved FFAs metabolism in rats hepatic tissue. ^23^ Nevertheless, green coffee extract administration has detrimental effects due to the caffeine content, ^24^^–^^26^ and the CGA antioxidant activity depends on the roasted level of green coffee beans. ^27^^,^^28^ Therefore, this study used decaffeinated light-roasted green coffee beans to avoid the detrimental effects of caffeine content and reach the highest antioxidant activity of CGA. Many studies showed that green tea or green coffee extract administration could treat metabolic syndrome. Still, analysis investigating the beneficial effect of these extract combinations in alleviating metabolic syndrome remains limited, whereas natural compounds have a more significant therapeutic effect when combined. ^29^ This study investigated the combination of green tea and decaffeinated, light roasted green coffee extract to improve free fatty acid-induced cardiac insulin resistance by improving the adiponectin/FAS pathways.

## Methods

### Ethics statement

All experimental procedures were approved by the ethical committee of Faculty of Medicine, Brawijaya University with registration number 148/EC/KEPK-S2/06/2021. All efforts were made to ameliorate harm to the animals by using the standard protocol from the Indonesian Ministry of Health ethical research guidelines for animal experimental research. ^30^

### Animals and experimental design

This study is part of a larger study ^31^ which used 25 males Sprague–Dawley rats (aged 9 weeks, weighed 230 to 340 gram) obtained from the National Agency of Drug and Food Control, Indonesia. The ethical committee approved the experimental protocols of the Faculty of Medicine, Brawijaya University. Rats were maintained and acclimatized in the environmentally controlled standard cage as described in our previous study. ^31^ Animals were grouped as the previous study as follows: i) normal control group (NC) that were assigned to standard chow (Indofeed™, Indonesia) without STZ injection; metabolic syndrome rats were obtained after HFHS diet and low dose STZ (bioWORLD cat.41910012-4) injection intraperitoneally at the second week of the protocol. NCEP ATP III criteria confirmed metabolic syndrome features. The metabolic syndrome rats were assigned to four groups ii) metabolic syndrome group (MS); iii) green tea group (GT) that were given the HFHS diet, STZ injection, and green tea extract (300 mg/kg b.w.) administration per oral; iv) green coffee group (GC) that were assigned to HFHS diet, STZ injection, and green coffee extract (200 mg/kg b.w.) administration; v) combination group (CM) that were assigned to HFHS diet, STZ injection, and combination of green tea (300 mg/kg b. w.) and green coffee (200 mg/kg b. w.) extract administration, respectively. ^32^ Green tea and coffee extract were administered in millilitres *via* oral gavage daily based on weekly measured body weight. ^32^ After 9 weeks of exact treatment, animals were euthanized by cervical decapitation previously anaesthetized using diethyl ether after a 12-hour fast. As soon as the rats died, the blood samples were obtained from the heart and transferred into a microcentrifuge tube to get serum samples by centrifugation (4,000 × *g* for 15 minutes at 4°C). ^32^

### Extraction procedures of green tea leaves

The green tea leaves were obtained from Sukawana, Bandung, Indonesia (1550 MAMSL). Protocol of green tea extraction followed the procedure of Saifur Rohman *et al.* (2021). ^32^

### Extraction procedures of decaffeinated, light roasted green coffee beans

The coffee beans used in this experiment were *Coffea canephora*/ *robusta.* The coffee beans were obtained from Dampit, Malang, Indonesia (800 MAMSL). The protocol of green coffee extraction followed the procedure of Saifur Rohman *et al*. (2021). ^32^

### High-performance liquid chromatography analysis

Bioactive compound levels in green tea extract (EGCG) and green coffee bean extract (caffeine and CGA) were analyzed by high-performance liquid chromatography (HPLC) system using a Shimadzu Brand chromatograph (model SCL10AVP, Japan) that set up with a C-18 reverse-phase column (Shim-pack VP ODS 5μm 150 × 4.6 mm). Details of the method were explained in the previous study. ^32^

### Dose determination

Our previous study determined the exact doses based on the optimal doses for each extract. ^32^

### Physiological measurements

Daily food intake and fluid intake were measured each day, and body weight every week. Foods and fluids intake were measured by subtracting the amount provided by the remaining. More information was written in the previous study. ^32^

### Biochemistry analysis

The fasting blood glucose (BIOLABO, cat no. 80009), triglycerides (TG) (cat no. 80019), and HDL-Cholesterol (BIOLABO, cat no. 86516) were analyzed with commercial kits (Biolabs, France) enzymatically as in the previous study. ^32^

### Enzyme-linked immunosorbent assay (ELISA)

Rat serum was collected at the end of the study and stored at −80 ^o^C. ELISA analysis was conducted for measuring non-esterified fatty acid (NEFA) (Ref E-BC-K014, Elabscience, United States), (Ref E-EL-R2466, Elabscience, United States), and adiponectin level (Ref E-EL-R3012, Elabscience, United States). The measurements were conducted using the manual protocol by the manufacturer and read using ELx808 Absorbance Microplate Reader (BioTek, China) to get the result as ng/mL.

### Homeostatic model assessment for insulin resistance (HOMA-IR)

This study measured HOMA-IR using the Commercial ELISA Kit (Ref E-EL-R2466, Elabscience, United States). The measurement was conducted using the manual protocol by the manufacturer and read using EL×808 Absorbance Microplate Reader (BioTek, China) to get the result as ng/mL. HOMA-IR was used to calculate an index from the product of the fasting concentrations of blood glucose (mg/dL) and insulin (microU/L) levels divided by 14.1. Lower levels of HOMA-IR indicate greater insulin sensitivity, and higher levels indicate insulin resistance. ^33^

### Systolic blood pressure measurements

Blood pressure was measured three times by a tail-cuff sphygmomanometer technique (Ugo Basile 58500) at the baseline and the end of the study. The average of those three measurements was calculated and shown as the final reading for SBP.

### Isolation of total RNA and reverse transcription-polymerase chain reaction

Total RNA was obtained from liver and heart tissues and isolated using the easy-BLUE (Intron Biotechnology, cat no. 17061). Reverse transcription reaction was converted by a ReverTra Ace-α kit (Toyobo, FSK-101). The mRNA expression levels were measured using the touchdown PCR protocol with polymerase chain reaction (PCR) LightCycler 96 system (Takara, cat no. TP600). The PCR process was performed using a GoTaq Green Master PCR Kit (Promega, cat no, M7822) according to the manufacturer's protocols with one of the target gene primers. The primer sequences were as follows: β-actin forward 5′-CGA GTA CAA CCT TCT TGC AG-3′, reverse 5′-CAT TGT AGA AAG TGT GGT GC-3′; FAS forward 5′-TGG AGA AGC CCA GGA ACA ACT CAT-3′, reverse 5′-ACC GAG TAA TGC CGT TCA GTT CCT-3′; Adipo-R1: forward 5′-GAC AGG CCT AGG TGT CCA TCA-3′, reverse 5′-TCG TAT GGG ATG ACC CTC CA-3′; PI3K forward 5′-CCT CTC CTT ATA AAG CTC CTG GAA-3′, reverse 5′-GAT CAC AAT CAA GAA GCT GTC GTA A-3′; IRS1 forward 5′-AAG CAC CTG GTG GCT CTC TA-3′, reverse 5′-TCA GGA TAA CCT GCC AGA CC-3′; IRS2 forward 5′-ATA CCG CCT ATG CCT GTC TG-3′, reverse 5′-AGA AGA AGC TGT CCG AGT GG-3′; PDK1 forward 5′-CGT CCC GCA CGT AGA G-3′, reverse 5′-TCC TCA GCA CTC TTG TCC TTA-3′; AKT forward 5′-TCA CCT CTG AGA CCG ACA CC-3′, reverse 5′-ACT GGC TAG TAG GAG AAC TGG-3′; GLUT4 forward 5′-CTT CCT TCT ATT TGC CGT CCT C-3′, reverse 5′-GCT GCT TTG TCC TTC ATC CTG-3′. Gene expression was defined as the relative expression level after being compared with the housekeeping gene (β-actin).

### Data and statistical analysis

Data were analyzed using SPSS 25 with independent t-test analysis with a confidence level of 95% as significant at p ≤ 0.05.

## Results

### The concentration of bioactive compounds in green tea and green coffee extract on HPLC analysis

HPLC analysis showed that EGCG concentration in green tea extract was 74.126 μg/g. Meanwhile, CGA, caffeine, and polyphenol concentrations in the green coffee extract was 27.134 μg/g and 43,473 μg/g, respectively. ^32^

### Biochemistry characterization in experimental animals

Our previous study reported that rats with the HFHS diet and low-dose STZ injection had metabolic syndrome. It was proved by the measurement of systolic blood pressure (SBP), fasting blood glucose (FBG) level, triglyceride (TG), and HDL cholesterol (HDL) plasma level in rats induced HFHS diet with STZ injection (MS) group met the metabolic syndrome characteristic in accordance to NCEP-ATP III criteria in 8 weeks of duration. Significant differences in SBP, FBG, TG, and HDL levels were observed between the normal control group (NC) and the metabolic syndrome group (MS) (p < 0.05). ^34^ The pre- and post-test in all extract-intervention groups showed improvement in SBP, FBG, TG, and HDL levels. Meanwhile, the GC group had no statistically significant difference between pre-and post-test, except the HDL level (data were available in https://doi.org/10.6084/m9.figshare.13249163.v3).

### A combination of green tea and decaffeinated, light roasted green coffee extract lowered plasma non-esterified free fatty acids (NEFA) levels

**Figure 1.**
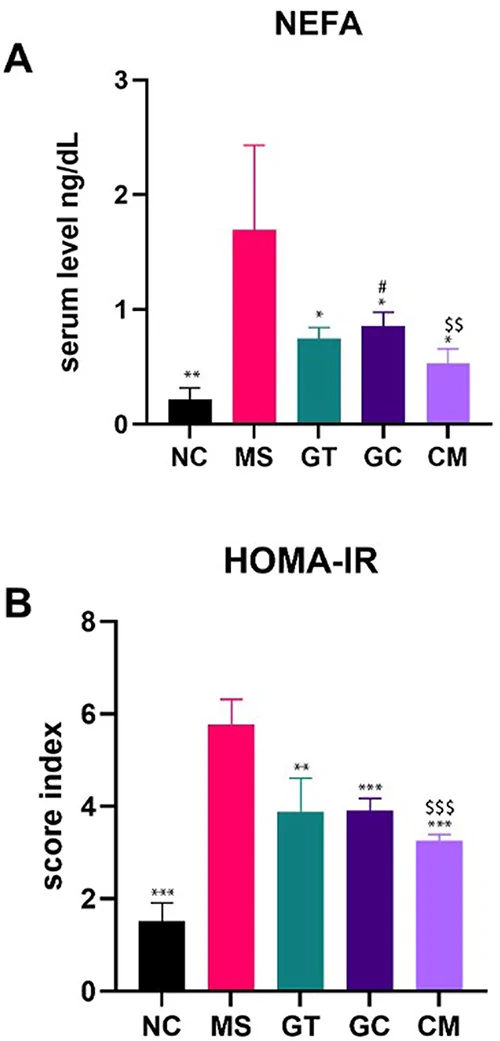
Free fatty acid levels and insulin resistance status. A. Serum level of non-esterified free fatty acids; B. HOMA-IR score index was used as representation of insulin resistance status. Data are expressed as mean ± SEM (N = 4-5). *p < 0.05, **p < 0.01, ***p < 0.001, ****p < 0.0001 compared with MS. #p < 0.05, ##p < 0.01, ###p < 0.001, ####p < 0.0001 compared with GT. $p < 0.05, $$p < 0.01, $$$p < 0.001 compared with GC. MS (metabolic syndrome), NC (normal chow), GT (green tea extract), GC (decaffeinated-light roasted green coffee extract), CM (combination of both extracts).

A significant increase was observed in plasma NEFA levels in the MS group compared tothe NC group (p < 0.05). After extract intervention, the plasma NEFA levels in all extract-treated rats were significantly lower (p < 0.05) compared to that of the MS group. Interestingly, the CM group had the least plasma NEFA levels and a significant difference than GT (p < 0.05) and GC (p < 0.01) was observed in plasma NEFA levels ( Figure 1A). It suggested that combining the green tea extract and decaffeinated light roasted green coffee was more effective in reducing FFAs than green tea or green coffee extract single administration ( Figure 1A).

### A combination of green tea and decaffeinated light roasted green coffee extract improved adiponectin levels and adipo-R1 gene expression

**Figure 2.**
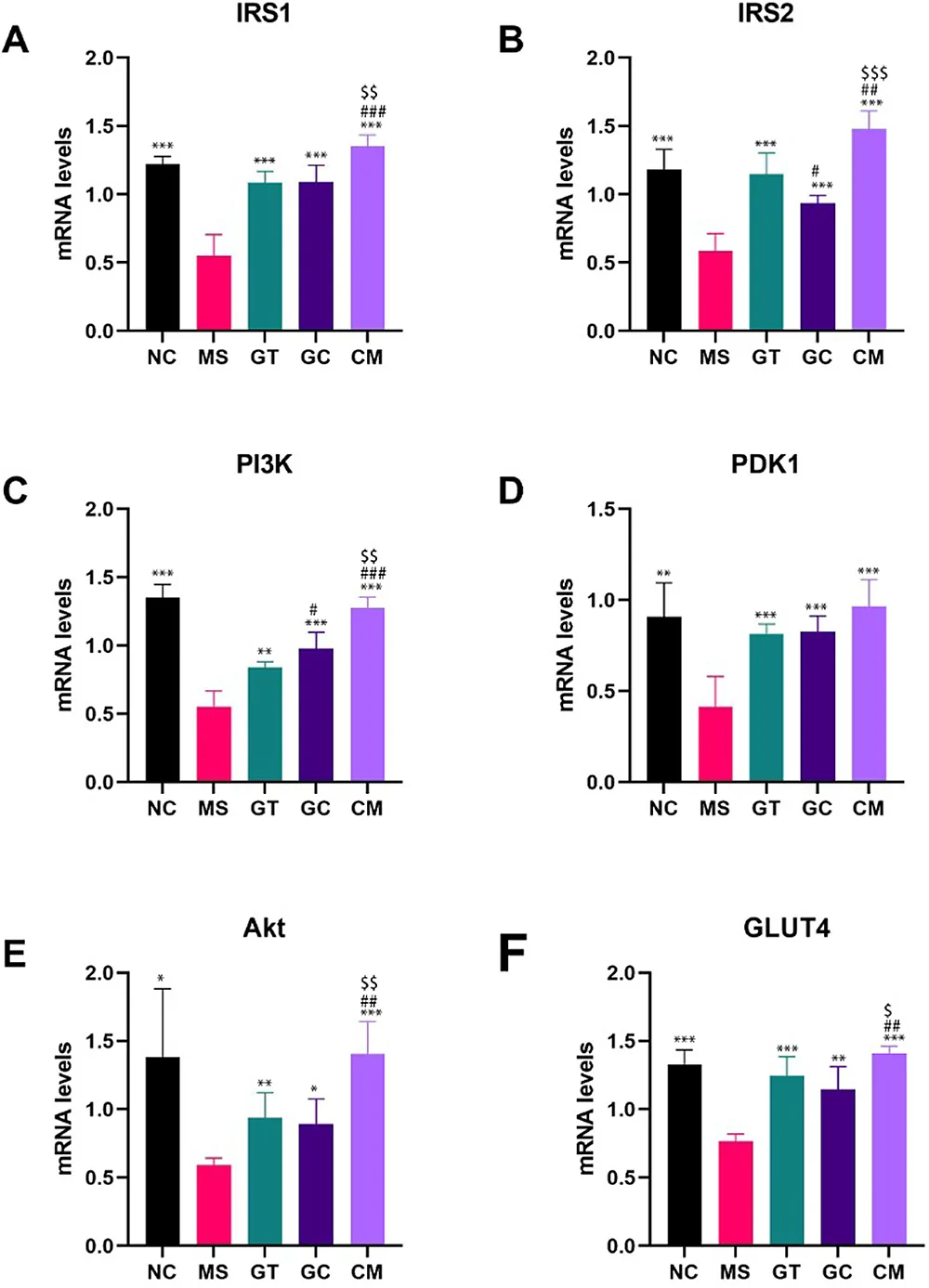
Adiponectin/FAS Measurement. A. Adiponectin serum levels; B. Gene expression of Adipo-R1; C. Gene expression of FAS. Data are expressed as mean ± SEM (N = 4-5). *p < 0.05, **p < 0.01, ***p < 0.001, ****p < 0.0001 compared with MS. #p < 0.05, ##p < 0.01, ###p < 0.001, ####p < 0.0001 compared with GT. $p < 0.05, $$p < 0.01, $$$p < 0.001 compared with GC. MS (metabolic syndrome), NC (normal chow), GT (green tea extract), GC (decaffeinated-light roasted green coffee extract), CM (combination of both extracts).

Adiponectin levels were significantly lower in the MS group than in the NC group (p < 0.05). The GT group showed lower levels of adiponectin than the GT group. Still, the combination extract group showed a better effect in improving adiponectin levels significantly compared to that of the GT and GC groups (p < 0.05) ( Figure 2A). Moreover, the relative mRNA expression levels of adiponectin-receptor 1 (Adipo-R1) in this study were also significantly lower in the MS group compared to that of the NC group (p < 0.001) ( Figure 2B). All extract-treated groups, either GT, GC, or CM, revealed a significantly higher relative mRNA expression in Adipo-R1 gene expression compared to that of the MS group (p < 0.001) ( Figure 2B). Moreover, the CM group showed the highest Adipo-R1 mRNA expression levels among other extract-treated groups (p < 0.01) ( Figure 2B).

### A combination of green tea and decaffeinated light roasted green coffee extract improved free fatty acids levels and FAS gene expressions

This study revealed the effect of green tea and green coffee extract administration on the free fatty acid synthesis pathways by analyzing the NEFA levels and adiponectin/Adipo-R1/AMPK/FAS pathways involvement.

There was an increase in fatty acid synthase (FAS) gene expression in the liver. The expression of relative mRNA level of FAS was significantly higher in the MS group compared to that of the NC group (p < 0.001) ( Figure 2C). All extract-treated groups showed a lower relative mRNA level of FAS gene in the GT, GC, or CM groups and significantly different from the MS group (p < 0.05) ( Figure 2C). However, there was no significant difference in FAS gene expression between the GT and GC groups (p > 0.05). Still, the FAS gene expression in the CM group showed a significant difference compared to the GT and GC group (p < 0.01) ( Figure 2C). These results were linear with our previous study; we reported that HFHS diet and low dose STZ injection-induced metabolic syndrome rats had lower AMPK-α2 gene expression (p < 0.05). Meanwhile, the combination of green tea and green coffee extract showed substantially higher AMPK-α2 expression compared to that of single extract either green tea or green coffee extract administration (p < 0.05). ^34^ Therefore, this study revealed that green tea or green coffee extract administration could ameliorate the FFAs levels by improving the adiponectin/adipo-R1/AMPK/FAS pathways in liver tissue rats. However, the combined extract was more effective.

### A combination of green tea and decaffeinated light roasted green coffee extract improved HOMA-IR

HFHS diet with STZ injection rats showed a higher homeostatic model assessment for insulin resistance (HOMA-IR) index compared to that of the NC group (p < 0.001) ( Figure 1B). All of the extract-treated groups revealed a lower HOMA-IR index compared to the MS group (p < 0.05) ( Figure 1B). Moreover, the CM group showed the lowest level in the HOMA-IR index among other extract-treated groups (p < 0.001). This indicated that the combination revealed a better effect in improving insulin resistance.

### A combination of green tea and decaffeinated light roasted green coffee extract ameliorated cardiac insulin resistance marker expressions

**Figure 3.**
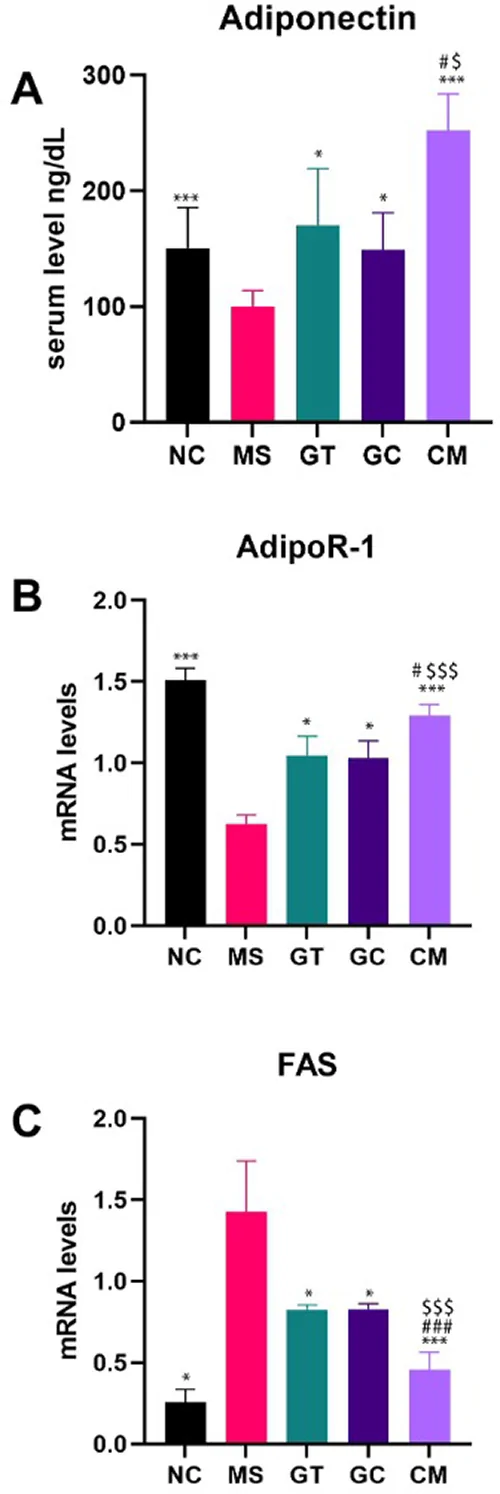
Gene expression measurement of cardiac insulin signalling. A. Relative mRNA expression level of IRS1; B. Relative mRNA expression level of IRS2; C. Relative mRNA expression level of PI3K; D. Relative mRNA expression level of PDK1; E. Relative mRNA expression level of Akt; F. Relative mRNA expression level of GLUT4. Data are expressed as mean ± SEM (N = 4-5). *p < 0.05, **p < 0.01, ***p < 0.001, ****p < 0.0001 compared with MS. #p < 0.05, ##p < 0.01, ###p < 0.001, ####p < 0.0001 compared with GT. $p < 0.05, $$p < 0.01, $$$p < 0.001 compared with GC. MS (metabolic syndrome), NC (normal chow), GT (green tea extract), GC (decaffeinated-light roasted green coffee extract), CM (combination of both extracts).

The higher HOMA-IR in HFHS diet with low dose STZ injection-induced rats was accompanied by a decrease of cardiac insulin signalling protein gene expression compared to that of the NC group (p < 0.05) ( Figure 1B). It was illustrated by the lower expression of IRS1/2, PI3K, PDK1, Akt, and GLUT4. A higher cardiac insulin signalling protein gene expression was observed in all extract-treated groups. Nevertheless, the GT group showed higher IRS2, AKT, and GLUT4 gene expressions compared to that of the GC group ( Figure 3B, E, F). However, those markers’ relative mRNA expression levels were higher in the CM group than in the GT or GC group (p < 0.001) ( Figure 3).

## Discussion

Metabolic syndrome is a metabolic disturbance characterized by insulin resistance and a decrease of adiponectin as a biomarker. Adiponectin has essential roles in metabolic profile, such as improved glucose and lipid metabolism and insulin resistance. ^35^ Adiponectin, an inducer of AMP-activated protein kinase (AMPK) has a regulatory effect on glucose and lipid metabolism that had an essential role in free fatty acid production through inhibition of fatty acid synthetase (FAS). ^36^ The present study showed lower adiponectin and higher FFA levels in MS rats than the normal rats and intervention rat groups. The administration of green coffee and green tea in MS rats improved adiponectin and FFA serum levels. The improvement of adiponectin and FFA levels were related to the modulation of adipo-R1 and AMPK gene expression in hepatic tissue. ^37^^–^^39^ This study showed improvement in adipoR1 in the extract-treated groups. Additionally, FAS gene expression in hepatic tissue was lower in either the green tea or green coffee group. Furthermore, our previous study showed that hepatic AMPK expression was improved in all intervention groups, and the highest level showed in the combination extract group. ^34^

Lower adiponectin levels and higher FFA levels are related to insulin resistance in hepatic, skeletal, and cardiac tissue. ^35^^,^^40^ This study showed that higher HOMA-IR levels reflected insulin resistance conditions. The highest HOMA-IR was shown in MS group rats compared to that of intervention group rats. Moreover, green tea and green coffee extract administration had significantly lower HOMA-IR levels compared to that of MS group rats. Lower HOMA-IR in the extract-treated group was accompanied by improvement of insulin signalling in cardiac tissue. Our study showed cardiac tissue gene expression improvement related to insulin signalling, such as through IRS, Akt, PI3K, PDK1, and GLUT4 in MS rat with green tea and green coffee administration. The results showed that insulin signaling gene expressions were significantly higher in the intervention group compared to MS group rats. Moreover, the combination of green tea and green coffee had the highest expressions.

The present study aimed to investigate the effect of combining green tea and green coffee on cardiac insulin resistance caused by the elevation of FFA serum and lower adiponectin levels. This study showed that the administration of green tea and green coffee improved cardiac insulin resistance by reducing serum free fatty acids levels and elevating serum adiponectin levels by modulation of adipo-R1, AMPK, and FAS gene expressions. These results were similar to previous studies that improving serum FFA and adiponectin signalling pathways could attenuate insulin resistance. ^41^^–^^44^ Although animal and human studies have shown that lowering serum FFAs directly improved insulin resistance in some tissues such as muscles, adipose, hepatic, and endothelial tissues, few studies showed the effect on cardiac tissues. Moreover, green coffee administration could improve blood lipids and increase metabolic rates, fatty acid oxidation, and hepatic triglyceride in obese animal rat or mice models using diet or genetic modification. ^12^^,^^45^^–^^47^ In addition, a chlorogenic acid compound in the green coffee extract was associated with the improvement of adiponectin levels and had an important role in insulin sensitivity and inflammation. ^48^ Besides, green tea administration was known to have similar effects. ^49^ Li *et al.* (2018) study showed that the EGCG compound in green tea extract inhibited gene expression involved in synthesizing de novo fatty acids, such as FAS, ACC, and SC. ^50^ Other studies showed that EGCG had a beneficial effect on insulin resistance by improving the AMPK pathways. ^18^

Administration of a combination of green coffee and green tea extract in this study showed a higher adiponectin level compared to that of green coffee or green tea single extract administration. Additionally, the Adipo-R1 gene expression was higher in the CM group. Previous studies showed that green tea extract had a beneficial effect on the adiponectin signalling pathways through improving adiponectin levels and modulating adipo-R1 expressions. ^37^^,^^38^ On the other hand, few studies have revealed the effect of green coffee on the improvement of FFA levels. ^51^^,^^52^ Our study were also showed that green tea and green coffee administration were adequate to reduce serum FFA levels. This study was in accordance with previous studies that revealed the improvement of adiponectin and adipo-R1 is involved in lower serum FFA levels. ^18^^,^^41^^,^^45^^–^^47^ Interestingly, the combination of both extracts exhibited lower serum FFA levels than green tea or decaffeinated-light roasted green coffee alone.

Elevation of serum FFA levels is caused by excess nutrition intake, adipose lipolysis, and de novo fatty acid synthesis (glucose utilization), mainly in the hepatic tissues. ^39^ The present study showed that hepatic de novo fatty acid synthesis was increased in metabolic syndrome rats. It was illustrated by increasing FAS mRNA expressions in the MS group (P < 0.01 vs. NC group). This finding was linear with our previous study that AMPK mRNA expressions were reduced in MS rats. ^34^ AMPK has an inhibitory effect on FAS ^53^; thus, the reduction of AMPK showed a decrease in inhibitory regulation effect. Nevertheless, green tea or green coffee intervention in the present study showed a decrease in FAS expressions, and these were similar to other previous studies ^45^^,^^46^^,^^51^^,^^54^^,^^55^; however, our findings showed no significant difference between green tea and green coffee extract rat groups. Meanwhile, the combination of light roasted decaffeinated green coffee and green tea extract significantly reduced FAS mRNA expression compared to that of single extract groups. These findings showed that green tea and green coffee had more practical effects on improving hepatic de novo fatty acid synthesis, thus reducing serum FFAs levels.

It was known that FFA induced insulin resistance. ^44^^,^^56^ This study revealed HOMA-IR accompanied the increase of serum FFA levels in MS rats. Compared with previous studies, ^57^^–^^59^ we had similar findings that administration of green tea decaffeinated-light roasted green coffee had beneficial effects on improving insulin resistance. It was illustrated by a lowering of HOMA-IR in all intervention groups. Meanwhile, the administration of the combination of both extracts showed a lower level of HOMA-IR than green tea or green coffee administration alone. A previous study showed that the administration of EGCG ^54^^,^^60^ attenuated insulin sensitivity in skeletal and adipose tissue.. Moreover, CGA and other polyphenols in green coffee improved insulin sensitivity in HFD Mice. ^51^ HOMA-IR reflects insulin resistance conditions from multi organs such as adipose, heart, and muscle, including skeletal and cardiac muscle. ^61^^,^^62^

Many reviews have revealed that FFA could induce alteration of cardiac structure and function without coronary disease or hypertension, ^7^ which is called diabetic cardiomyopathy, and insulin resistance is the early step in the development of the disease. ^63^ Many studies suggest that an increase of serum FFA levels can induce insulin resistance by inhibiting IRS1/PI3K/Akt in cardiac muscle, ^64^^–^^66^ and associated with cardiac remodelling and dysfunction. ^67^^,^^68^ It was recently known that FFAs could interact with toll-like receptors (TLR) and induce insulin resistance through the disturbance of insulin signalling pathways in cardiac tissues. ^11^^,^^69^ Our findings observed that improvement of serum FFA levels was accompanied by improved cardiac insulin signalling pathways. All treatment groups showed a high of IRS1/2, PI3K, PDK1, Akt, and GLUT4 mRNA expression, but the GT group showed higher IRS2, Akt, and GLUT4 than the GC group. Rebollo-Hernanz *et al*. (2019) recently showed that phenolic compounds in green coffee, especially CGA, modulate adipogenesis and insulin resistance via PI3K/Akt signalling pathways in adipocytes. ^70^ Another study showed that chlorogenic acid stimulates glucose transport in skeletal muscle *via* AMPK activation in db/db mice. ^58^ Besides, the green tea administration showed the ameliorating mechanism of hyperglycemia by promoting GLUT4 translocation in skeletal muscle of diabetic rodents. ^60^ Moreover, another study revealed that EGCG administration induced GLUT4 translocation in skeletal muscle through PI3K- and AMPK-dependent pathways. ^16^ Nevertheless, the combination of green tea and decaffeinated-light roasted green coffee extract had the highest expressions. It showed that green tea and green coffee extract had synergistic interaction that consequently improved insulin sensitivity.

This study had several limitations. Firstly, we did not investigate the biomarker in adipose tissues, which might give a comprehensive understanding of green tea and green coffee administration’s effect on lipid metabolism. Secondly, we could not exhibit the molecular mechanisms for the effects of green tea and green coffee combination administration.

## Conclusion

Our study revealed clear evidence that a combination of green tea and decaffeinated light roasted green coffee extracts improved cardiac insulin resistance better than single extract administration by ameliorating free fatty acids through adiponectin/FAS pathways modulation.

## Data availability

Figshare: Underlying data for ‘Green Tea and decaffeinated light roasted green coffee extract combination improved cardiac insulin resistance through free fatty acids and adiponectin/FAS pathways amelioration in metabolic syndrome rat model’ https://doi.org/10.6084/m9.figshare.13249163.v3.

This project contains the following underlying data:

- Data of Metabolic Syndrome Rat Model.xlsx (This file contains the analyzed data)
- RAW data metabolic syndrome.xlsx (This file contains the actual observed values of the variables)
- Chlorogenic acid Coffee HPLC.pdf (This file contains the value of coffee chlorogenic acid levels as measured by HPLC)
- Caffeine coffee HPLC.pdf (This file contains the value of coffee caffeine levels as measured by HPLC)
- Green Tea Catechin HPLC.pdf (This file contains the value of the catechin levels in the tea)

Data are available under the terms of the Creative Commons Attribution 4.0 International license (CC-BY 4.0).

## Author contributions

M.L: Conceptualization, Data Curation, Methodology, Writing – Review & Editing

M.S.R: Conceptualization, Funding acquisition, Supervision, Writing – Review & Editing

D.A.N: Conceptualization, Data Curation, Methodology, Resources

M.N.R: Data Curation, Formal Analysis, Investigation, Writing – Original Draft Preparation

N.A.W: Data Curation, Investigation, Writing – Original Draft Preparation

N.W: Project Administration, Supervision, Writing – Review & Editing
