# Green Tea and Decaffeinated Light Roasted Green Coffee Extract Combination Improved Cardiac Insulin Resistance through Free Fatty Acids and Adiponectin/FAS Pathways Amelioration in Metabolic Syndrome Rat Model

**DOI:** 10.12688/f1000research.55470.3

**Published:** 2026-07-03

**Authors:** Mifetika Lukitasari, Mohammad Saifur Rohman, Dwi Adi Nugroho, Mukhamad Nur Kholis, Nila Aisyah Wahyuni, Nashi Widodo

**Affiliations:** 1 Department of Nursing, Faculty of Medicine, Brawijaya University, Malang, East java, +62, Indonesia; 2 Department of Cardiology and Vascular Medicine, Faculty of Medicine, Brawijaya University, Malang, East java, +62, Indonesia; 3 Department of Herbal Medicine, Brawijaya Cardiovascular Research Group, Faculty of Medicine, Brawijaya University, Malang, East java, +62, Indonesia; 4 Magister of Biomedical Sciences, Faculty of Medicine, Brawijaya University, Malang, East java, +62, Indonesia; 5 Department of Biology Mathematics and Natural Sciences, Brawijaya, Malang, East java, +62, Indonesia

**Keywords:** metabolic syndrome; green tea; green coffee; cardiac insulin resistance

## Abstract

**Background:**

Insulin resistance has been independently associated with cardiac diseases. Free fatty acids (FFAs) are known to induce cardiac insulin resistance via low-grade inflammation. Therefore, lowering FFA levels may improve cardiac insulin resistance. This study investigated the effects of a combination of green tea and decaffeinated light-roasted green coffee extract on free fatty acid-induced cardiac insulin resistance by modulating the adiponectin/FAS pathways.

**Methods:**

This study used 25 male Sprague-Dawley rats. Metabolic syndrome (MS) was induced using a high-fat, high-sucrose (HFHS) diet and a low-dose streptozotocin (STZ) injection, while a normal chow (NC) diet served as the healthy control. The MS rats were treated for 9 weeks with green tea (300 mg/kg b.w.), decaffeinated light-roasted green coffee (200 mg/kg b.w.), or a combination of both extracts. The experimental subjects were divided into five groups: 1) MS (HFHS diet + STZ), 2) NC (normal chow), 3) GT (green tea extract), 4) GC (decaffeinated light-roasted green coffee extract), and 5) CM (combination of both extracts). Adiponectin and HOMA-IR levels were analysed using ELISA, while the gene expression of Adipo-R1, FAS, PI3K, PDK1, Akt, and GLUT4 was measured using RT-PCR.

**Results:**

The combination of green tea and decaffeinated light-roasted green coffee demonstrated synergistic effects in reducing FFA levels. The adiponectin/FAS pathways were attenuated in the CM group. Furthermore, the combination therapy improved cardiac insulin resistance marker expressions, including IRS-1/2, PI3K, PDK1, Akt, and GLUT4.

**Conclusions:**

The combination of green tea and decaffeinated light-roasted green coffee extract improved cardiac insulin resistance more effectively than the administration of either extract alone, by reducing FFA levels through the modulation of the adiponectin/FAS pathways.

## Editorial note

Editorial Note (4 ^th^ August 2023): The F1000 Editorial Team has not yet received a new version of this article, as detailed in the Editorial Note published on 16 ^th^ June 2023. The F1000 Editorial Team is actively contacting the authors to request the new version of the article. Peer review activity remains suspended until the authors publish a new version of this article.

Editorial Note (16 ^th^ June 2023): Since publication, it has been brought to the attention of the Editorial Team that the article was missing key information regarding animal treatment and ethical approval. The Editorial Team requested further detail and an explanation from the authors in March 2023. The authors provided an adequate response and were requested by the Editorial Team to create a new version of the article to include the additional details. Peer review activity has been suspended until the authors publish a new version of this article.

## Introduction

The incidence of metabolic syndrome (MS) features, such as obesity, hyperglycaemia, and insulin resistance, has increased, largely associated with excessive food consumption. ^1^ The prevalence of MS has risen by approximately 50–75% over the last decade. ^2^ Previous studies have established insulin resistance as a significant predictor and major feature of heart failure (HF). ^3, 4^ Research has further demonstrated that insulin resistance may contribute to cardiac remodelling, including hypertrophy, fibrosis, and cardiac dysfunction, even in the absence of coronary artery disease or hypertension. ^5– 9^ Lipotoxicity caused by free fatty acids (FFAs) is a primary driver of insulin resistance through low-grade inflammation in cardiac tissue. ^10, 11^ It reported that FFAs induce inflammation and suppress glucose uptake by inhibiting IRS-1 in cardiac tissue. ^12^ Conversely, reducing plasma FFA levels has been shown to improve cardiac function in high-fat diet-induced obese rat models. ^13^ Therefore, improving the lipid profile may prevent the development of cardiac insulin resistance.

Recently, natural compound derivatives have revealed beneficial effects in alleviating MS. One widely consumed compound is epigallocatechin-3-gallate (EGCG), which is primarily derived from green tea leaves. ^14^ Numerous studies have investigated the beneficial effects of green tea and its constituents in mitigating MS. Most findings indicate that green tea administration modulates insulin sensitivity, reduces blood glucose levels, and improves lipid profiles in animal models of metabolic syndrome. ^15– 17^ Additionally, FFA-induced insulin resistance and hyperglycemia-induced cardiac fibrosis can be attenuated by *in vivo* EGCG administration. ^18^

Furthermore, chlorogenic acid (CGA), a phenolic compound found in green coffee beans, has shown potential in alleviating MS. ^19, 20^ CGA potentiates insulin activity similar to the therapeutic mechanism of metformin, ^21^ without inducing obesity—an adverse effect often associated with thiazolidinedione (TZD) or insulin therapy. ^22^ Previous studies have also shown that CGA improves FFA metabolism in rat hepatic tissue. ^23^ Nevertheless, green coffee extract administration can have detrimental effects due to its caffeine content, ^24– 26^ and the antioxidant activity of CGA is dependent on the roasting level of the green coffee beans. ^27, 28^ Therefore, this study utilized decaffeinated, light-roasted green coffee beans to avoid the adverse effects of caffeine while maximizing the antioxidant activity of CGA.

Although many studies have demonstrated the efficacy of green tea or green coffee extract individually in treating metabolic syndrome, research investigating the combined effects of these extracts remains limited. Combining natural compounds often yields more significant therapeutic effects. ^29^ Consequently, this study investigated the combination of green tea and decaffeinated, light-roasted green coffee extract to improve FFA-induced cardiac insulin resistance through the modulation of the adiponectin/FAS pathways.

## Methods

### Ethics statement

All experimental procedures were approved by the Research Ethics Committee of the Faculty of Medicine, Universitas Brawijaya (Registration Number: 148/EC/KEPK-S2/06/2021). All procedures were conducted in strict accordance with the guidelines provided by the Indonesian Ministry of Health for the ethical use of animals in research. Every effort was made to minimise pain, distress, and discomfort for the animals throughout the experimental period. ^30^

### Animals and experimental design

This study is part of a larger research project using 25 male Sprague–Dawley rats (aged 9 weeks, weighing 230–340 g) obtained from the National Agency of Drug and Food Control, Indonesia. The experimental protocols were approved by the Research Ethics Committee of the Faculty of Medicine, Universitas Brawijaya (Registration Number: 148/EC/KEPK-S2/06/2021). Rats were housed and acclimatised in an environmentally controlled standard cage, as previously described. ^31^

The animals were divided into five groups: a normal control group (NC), a metabolic syndrome group (MS), a green tea group (GT), a green coffee group (GC), and a combination group (CM). The NC group was fed with a standard commercial rat pellet diet (Indofeed™, Indonesia) without STZ injection. To induce metabolic syndrome, the MS, GT, GC, and CM groups were fed a high-fat, high-sucrose (HFHS) diet and induced with a low-dose streptozotocin (STZ; bioWORLD, cat. #41910012-4) intraperitoneal injection at the second week of the protocol. The HFHS diet was prepared by powdering the standard rat pellet diet and mixing it with 20% sucrose, 0.5% methionine, 2.5% salt, 2% monosodium glutamate, 15% egg yolks, and 20% white fat. ^31^ Metabolic syndrome features were confirmed according to NCEP ATP III criteria.

Following the induction, the treatment groups were managed as follows: the MS group received no further treatment; the GT group was administered green tea extract (300 mg/kg b.w.) orally; the GC group was administered decaffeinated light-roasted green coffee extract (200 mg/kg b.w.) orally; and the CM group was administered a combination of green tea (300 mg/kg b.w.) and decaffeinated light-roasted green coffee (200 mg/kg b.w.) extracts orally. The extracts were administered daily via oral gavage, with dosages adjusted weekly based on the rats’ body weight. ^32^ After 9 weeks of treatment, the animals were fasted for 12 hours and anesthetized with diethyl ether. Blood samples were collected directly from the heart (cardiac puncture), after which the animals were euthanized by cervical decapitation. The collected blood was centrifuged at 4,000 × g for 15 minutes at 4°C to obtain serum samples. The hearts were immediately harvested and preserved in an RNA buffer solution to maintain RNA integrity.

### Green tea extraction procedures

Green tea leaves were harvested from Sukawana, Bandung, Indonesia (1,550 m above mean sea level). Young green tea leaves were cleaned and dried using a cabinet dryer at 50°C until they reached 8–10% water content. The dried leaves were then boiled to produce a crude extract. The extract was filtered, and the resulting liquid phase was concentrated through rotary evaporation at 40°C (RV10 autoV, IKA). This concentrated extract was utilised for the treatment throughout the study. This method strictly adhered to the protocol established in a previous study. ^32^

### Green coffee extraction procedures

The coffee beans used in this experiment were Coffea canephora var. robusta, obtained from Dampit, Malang, Indonesia (800 m above mean sea level). The green coffee beans were light-roasted at 180–200°C (N500i) until they reached the “first crack” stage and then ground into a fine powder. The powder was subsequently extracted using ethanol. The resulting crude extract underwent filtration, followed by solvent removal using a rotary evaporator at 40°C (RV10 autoV, IKA) to obtain the concentrated extract. Detailed methodological steps followed established procedures from prior research. ^32^

### High-performance liquid chromatography analysis

Bioactive compound levels in green tea extract (EGCG) and green coffee bean extract (caffeine and CGA) were analysed by a high-performance liquid chromatography (HPLC) system using a Shimadzu brand chromatograph (model SCL10AVP, Japan) that was set up with a C-18 reverse-phase column (Shim-pack VP ODS 5 μm 150 × 4.6 mm). Details of the method were explained in the previous study. ^32^

### Dose determination

The dosages selected for this study were based on the optimal efficacy observed in previous research regarding green tea and decaffeinated light-roasted green coffee extract. Specifically, the green tea extract dosage of 300 mg/kg b.w. and the decaffeinated light-roasted green coffee extract dosage of 200 mg/kg b.w. were determined based on demonstrated therapeutic benefits in metabolic syndrome models as reported in previous studies. ^32^ These doses were chosen to maximize the synergistic potential of the bioactive compounds while ensuring the safety of the experimental animals.

### Physiological measurements

Daily food and fluid intake were recorded by subtracting the remaining amount from the total quantity provided. Body weight was monitored on a weekly basis. Further procedural details have been described in our previous work. ^32^

### Biochemistry analysis

Fasting blood glucose (BIOLABO, cat. #80009), triglycerides (TG) (cat. #80019), and HDL-cholesterol (BIOLABO, cat. #86516) were analyzed using commercial enzymatic kits (Biolabs, France), as previously documented. ^32^

### Enzyme-linked immunosorbent assay (ELISA)

Rat serum collected at the end of the experimental period was stored at −80°C. ELISA was performed to measure the levels of non-esterified fatty acids (NEFA) (Ref: E-BC-K014, Elabscience, USA) and adiponectin (Ref: E-EL-R3012, Elabscience, USA). All measurements were conducted according to the manufacturer’s manual protocols. The optical density was measured using an ELx808 Absorbance Microplate Reader (BioTek, China), and results were expressed in ng/mL.

### Homeostatic model assessment for insulin resistance (HOMA-IR)

The HOMA-IR index was calculated based on the product of fasting blood glucose (mg/dL) and fasting insulin (μU/mL) concentrations, divided by a constant of 14.1 (i.e., [Glucose × Insulin] /14.1). Serum insulin levels were measured using a commercial ELISA kit (Ref: E-EL-R2466, Elabscience, USA). All procedures strictly followed the manufacturer’s manual protocol, and absorbance was measured using an EL×808 Absorbance Microplate Reader (BioTek, China). Results for insulin were expressed in μU/mL. In the interpretation of these data, lower HOMA-IR values indicate higher insulin sensitivity, whereas higher values indicate the presence of insulin resistance. ^33^

### Systolic blood pressure measurements

Systolic blood pressure (SBP) was measured three times using a tail-cuff sphygmomanometer (Ugo Basile 58500) at both the baseline and the conclusion of the study. The final SBP reading for each subject was determined by calculating the average of these three measurements.

### Isolation of total RNA and reverse transcription-polymerase chain reaction

Total RNA was extracted from heart and liver tissues using the easy-BLUE reagent (Intron Biotechnology, cat. #17061). Reverse transcription was performed using the ReverTra Ace-α kit (Toyobo, FSK-101) to synthesize cDNA. Quantitative mRNA expression levels were subsequently measured using the touchdown PCR protocol on a LightCycler 96 system (Takara, cat. #TP600). The PCR amplification was conducted using the GoTaq Green Master PCR Kit (Promega, cat. #M7822) according to the manufacturer’s instructions.

The primer sequences were as follows:

β-actin forward 5′-CGA GTA CAA CCT TCT TGC AG-3′, reverse 5′-CAT TGT AGA AAG TGT GGT GC-3′; FAS forward 5′-TGG AGA AGC CCA GGA ACA ACT CAT-3′, reverse 5′-ACC GAG TAA TGC CGT TCA GTT CCT-3′; Adipo-R1: forward 5′-GAC AGG CCT AGG TGT CCA TCA-3′, reverse 5′-TCG TAT GGG ATG ACC CTC CA-3′; PI3K forward 5′-CCT CTC CTT ATA AAG CTC CTG GAA-3′, reverse 5′-GAT CAC AAT CAA GAA GCT GTC GTA A-3′; IRS1 forward 5′-AAG CAC CTG GTG GCT CTC TA-3′, reverse 5′-TCA GGA TAA CCT GCC AGA CC-3′; IRS2 forward 5′-ATA CCG CCT ATG CCT GTC TG-3′, reverse 5′-AGA AGA AGC TGT CCG AGT GG-3′; PDK1 forward 5′-CGT CCC GCA CGT AGA G-3′, reverse 5′-TCC TCA GCA CTC TTG TCC TTA-3′; AKT forward 5′-TCA CCT CTG AGA CCG ACA CC-3′, reverse 5′-ACT GGC TAG TAG GAG AAC TGG-3′; GLUT4 forward 5′-CTT CCT TCT ATT TGC CGT CCT C-3′, reverse 5′-GCT GCT TTG TCC TTC ATC CTG-3′.

Gene expression was defined as the relative expression level after being compared with the housekeeping gene (β-actin).

### Data and statistical analysis

All data were presented as mean ± standard error of the mean (SEM) using GraphPad Prism 8.3.1 (GraphPad Software, San Diego, CA, USA). Statistical analysis was performed using IBM SPSS Statistics for Windows, Version 25 (IBM Corp., Armonk, NY, USA). To determine the differences between specific experimental groups, independent t-tests were conducted for each pairwise comparison. Differences were considered statistically significant at *p* < 0.05.

## Results

### The concentration of bioactive compounds in green tea and decaffeinated light-roasted green coffee extract on HPLC analysis

HPLC analysis showed that EGCG concentration in green tea extract was 74.126 μg/g. Meanwhile, CGA, caffeine, and polyphenol concentrations in the green coffee extract was 27.134 μg/g and 43,473 μg/g, respectively. ^32^

### Biochemistry characterization in experimental animals

Our previous study reported that rats with the HFHS diet and low-dose STZ injection had metabolic syndrome. It was proved by the measurement of systolic blood pressure (SBP), fasting blood glucose (FBG) level, triglyceride (TG), and HDL cholesterol (HDL) plasma level in rats induced HFHS diet with STZ injection (MS) group met the metabolic syndrome characteristic in accordance to NCEP-ATP III criteria in 8 weeks of duration. Significant differences in SBP, FBG, TG, and HDL levels were observed between the normal control group (NC) and the metabolic syndrome group (MS) (p < 0.05). ^34^ The pre- and post-test in all extract-intervention groups showed improvement in SBP, FBG, TG, and HDL levels. Meanwhile, the GC group had no statistically significant difference between pre-and post-test, except the HDL level (data were available in https://doi.org/10.6084/m9.figshare.13249163.v3).

### A combination of green tea and decaffeinated light-roasted green coffee extract lowered plasma non-esterified free fatty acids (NEFA) levels

**Figure 1.**
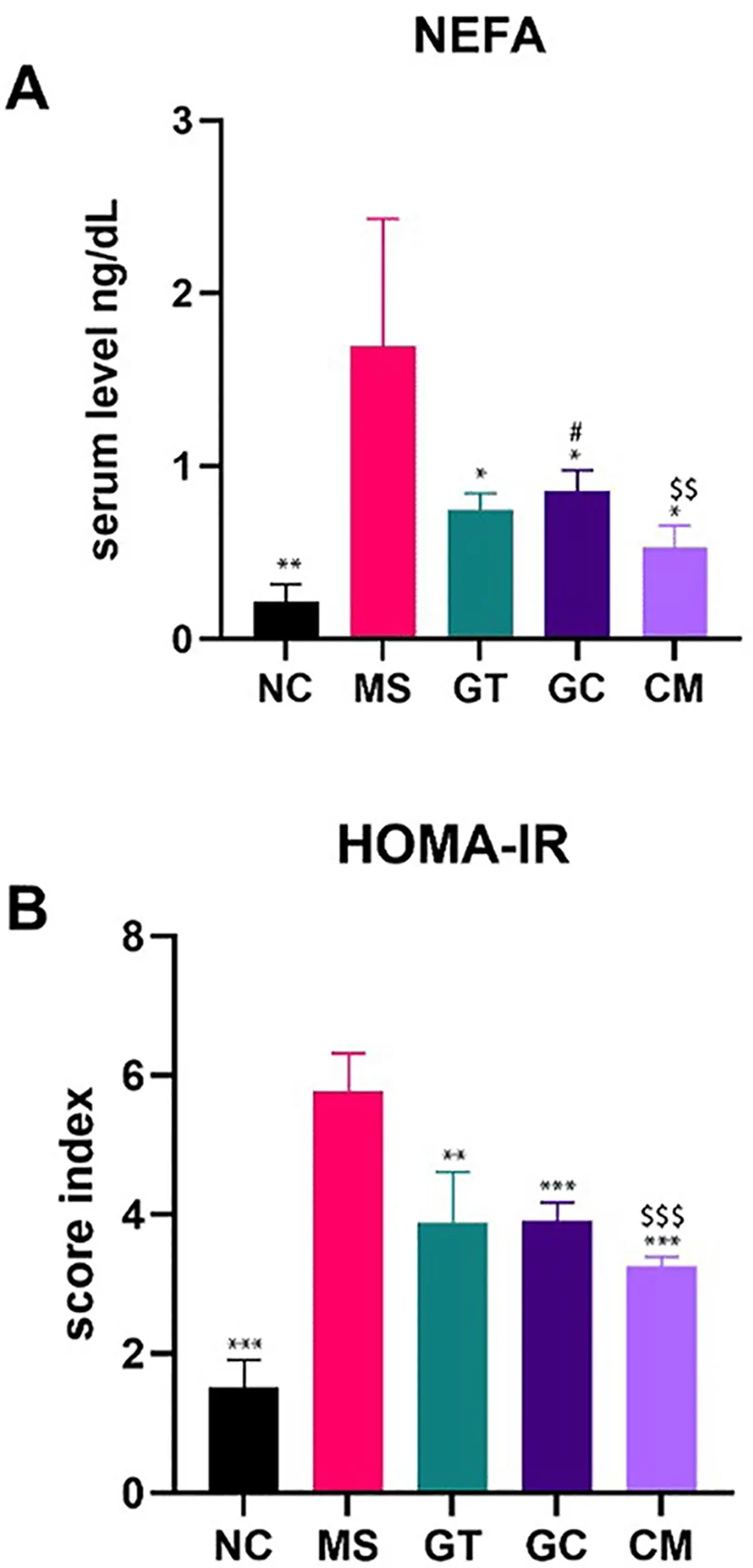
Free fatty acid levels and insulin resistance status. A. Serum level of non-esterified free fatty acids; B. HOMA-IR score index was used as representation of insulin resistance status. Data are expressed as mean ± SEM (N = 4-5). *p < 0.05, **p < 0.01, ***p < 0.001, ****p < 0.0001 compared with MS. #p < 0.05, ##p < 0.01, ###p < 0.001, ####p < 0.0001 compared with GT. $p < 0.05, $$p < 0.01, $$$p < 0.001 compared with GC. MS (metabolic syndrome), NC (normal chow), GT (green tea extract), GC (decaffeinated light-roasted green coffee extract), CM (combination of both extracts).

A significant increase was observed in plasma NEFA levels in the MS group compared to the NC group (p < 0.05). After extract intervention, the plasma NEFA levels in all extract-treated rats were significantly lower (p < 0.05) compared to that of the MS group. Interestingly, the CM group had the least plasma NEFA levels and a significant difference than GT (p < 0.05) and GC (p < 0.01) was observed in plasma NEFA levels ( Figure 1A). It suggested that combining the green tea extract and decaffeinated light roasted green coffee was more effective in reducing FFAs than green tea or green coffee extract single administration ( Figure 1A).

### A combination of green tea and decaffeinated light-roasted green coffee extract improved adiponectin levels and adipo-R1 gene expression

**Figure 2.**
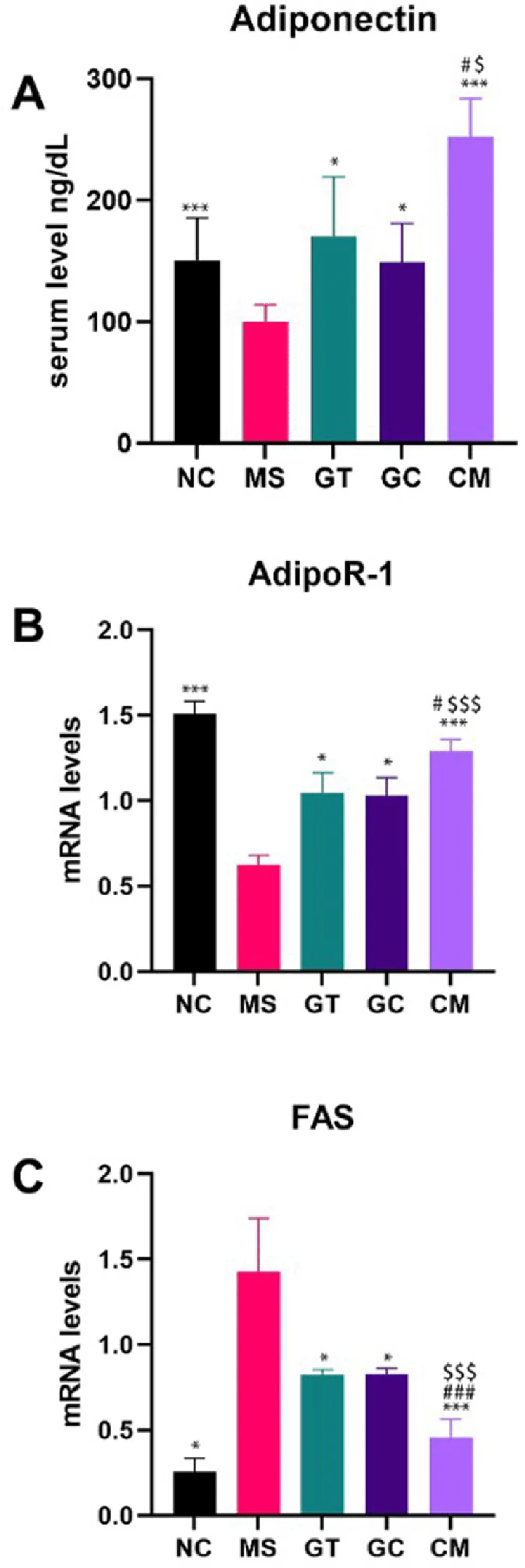
Adiponectin/FAS Measurement. A. Adiponectin serum levels; B. Gene expression of Adipo-R1; C. Gene expression of FAS. Data are expressed as mean ± SEM (N = 4-5). *p < 0.05, **p < 0.01, ***p < 0.001, ****p < 0.0001 compared with MS. #p < 0.05, ##p < 0.01, ###p < 0.001, ####p < 0.0001 compared with GT. $p < 0.05, $$p < 0.01, $$$p < 0.001 compared with GC. MS (metabolic syndrome), NC (normal chow), GT (green tea extract), GC (decaffeinated light-roasted green coffee extract), CM (combination of both extracts).

Adiponectin levels were significantly lower in the MS group than in the NC group (p < 0.05). The GT group showed lower levels of adiponectin than the GT group. Still, the combination extract group showed a better effect in improving adiponectin levels significantly compared to that of the GT and GC groups (p < 0.05) ( Figure 2A). Moreover, the relative mRNA expression levels of adiponectin-receptor 1 (Adipo-R1) in this study were also significantly lower in the MS group compared to that of the NC group (p < 0.001) ( Figure 2B). All extract-treated groups, either GT, GC, or CM, revealed a significantly higher relative mRNA expression in Adipo-R1 gene expression compared to that of the MS group (p < 0.001) ( Figure 2B). Moreover, the CM group showed the highest Adipo-R1 mRNA expression levels among other extract-treated groups (p < 0.01) ( Figure 2B).

### A combination of green tea and decaffeinated light-roasted green coffee extract improved free fatty acids levels and FAS gene expressions

This study revealed the effect of green tea and green coffee extract administration on the free fatty acid synthesis pathways by analyzing the NEFA levels and adiponectin/Adipo-R1/AMPK/FAS pathways involvement.

There was an increase in fatty acid synthase (FAS) gene expression in the liver. The expression of relative mRNA level of FAS was significantly higher in the MS group compared to that of the NC group (p < 0.001) ( Figure 2C). All extract-treated groups showed a lower relative mRNA level of FAS gene in the GT, GC, or CM groups and significantly different from the MS group (p < 0.05) ( Figure 2C). However, there was no significant difference in FAS gene expression between the GT and GC groups (p > 0.05). Still, the FAS gene expression in the CM group showed a significant difference compared to the GT and GC group (p < 0.01) ( Figure 2C). These results were linear with our previous study; we reported that HFHS diet and low-dose STZ injection-induced metabolic syndrome rats had lower AMPK-α2 gene expression (p < 0.05). Meanwhile, the combination of green tea and green coffee extract showed substantially higher AMPK-α2 expression compared to that of single extract, either green tea or green coffee extract administration (p < 0.05). ^34^ Therefore, this study revealed that green tea or green coffee extract administration could ameliorate the FFAs levels by improving the adiponectin/adipo-R1/AMPK/FAS pathways in liver tissue of rats. However, the combined extract was more effective.

### A combination of green tea and decaffeinated light-roasted green coffee extract improved HOMA-IR

HFHS diet with STZ injection rats showed a higher homeostatic model assessment for insulin resistance (HOMA-IR) index compared to that of the NC group (p < 0.001) ( Figure 1B). All of the extract-treated groups revealed a lower HOMA-IR index compared to the MS group (p < 0.05) ( Figure 1B). Moreover, the CM group showed the lowest level in the HOMA-IR index among other extract-treated groups (p < 0.001). This indicated that the combination revealed a better effect in improving insulin resistance.

### A combination of green tea and decaffeinated light-roasted green coffee extract ameliorated cardiac insulin resistance marker expressions

**Figure 3.**
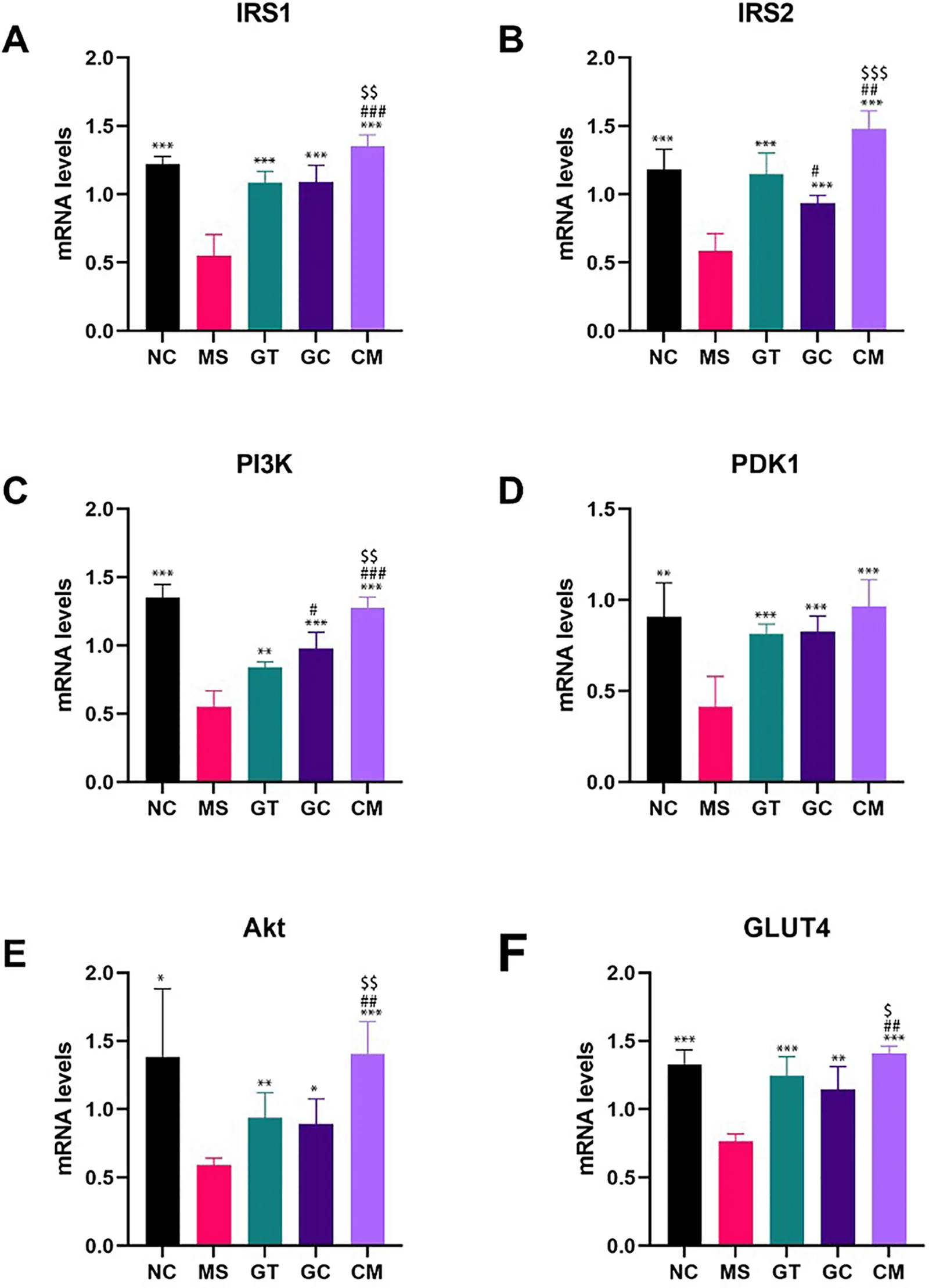
Gene expression measurement of cardiac insulin signalling. A. Relative mRNA expression level of IRS1; B. Relative mRNA expression level of IRS2; C. Relative mRNA expression level of PI3K; D. Relative mRNA expression level of PDK1; E. Relative mRNA expression level of Akt; F. Relative mRNA expression level of GLUT4. Data are expressed as mean ± SEM (N = 4-5). *p < 0.05, **p < 0.01, ***p < 0.001, ****p < 0.0001 compared with MS. #p < 0.05, ##p < 0.01, ###p < 0.001, ####p < 0.0001 compared with GT. $p < 0.05, $$p < 0.01, $$$p < 0.001 compared with GC. MS (metabolic syndrome), NC (normal chow), GT (green tea extract), GC (decaffeinated light-roasted green coffee extract), CM (combination of both extracts).

The higher HOMA-IR in HFHS diet with low-dose STZ injection-induced rats was accompanied by a decrease of cardiac insulin signalling protein gene expression compared to that of the NC group (p < 0.05) ( Figure 1B). It was illustrated by the lower expression of IRS1/2, PI3K, PDK1, Akt, and GLUT4. A higher cardiac insulin signalling protein gene expression was observed in all extract-treated groups. Nevertheless, the GT group showed higher IRS2, AKT, and GLUT4 gene expressions compared to that of the GC group ( Figure 3B, E, F). However, those markers’ relative mRNA expression levels were higher in the CM group than in the GT or GC group (p < 0.001) ( Figure 3).

## Discussion

Metabolic syndrome is a metabolic disturbance characterised by insulin resistance and a decrease of adiponectin as a biomarker. Adiponectin has essential roles in metabolic profiles, such as improved glucose and lipid metabolism and insulin resistance. ^35^ Adiponectin, an inducer of AMP-activated protein kinase (AMPK), has a regulatory effect on glucose and lipid metabolism that had an essential role in free fatty acid production through inhibition of fatty acid synthetase (FAS). ^36^ The present study showed lower adiponectin and higher FFA levels in MS rats than the normal rats and intervention rat groups. The administration of green coffee and green tea in MS rats improved adiponectin and FFA serum levels. The improvement of adiponectin and FFA levels was related to the modulation of adipo-R1 and AMPK gene expression in hepatic tissue. ^37^^–^^39^ This study showed improvement in adipoR1 in the extract-treated groups. Additionally, FAS gene expression in hepatic tissue was lower in either the green tea or green coffee group. Furthermore, our previous study showed that hepatic AMPK expression was improved in all intervention groups, and the highest level was shown in the combination extract group. ^34^

Lower adiponectin levels and higher FFA levels are related to insulin resistance in hepatic, skeletal, and cardiac tissue. ^35^^,^^40^ This study showed that higher HOMA-IR levels reflected insulin resistance conditions. The highest HOMA-IR was shown in MS group rats compared to that of intervention group rats. Moreover, green tea and green coffee extract administration had significantly lower HOMA-IR levels compared to those of MS group rats. Lower HOMA-IR in the extract-treated group was accompanied by improvement of insulin signalling in cardiac tissue. Our study showed cardiac tissue gene expression improvement related to insulin signalling, such as through IRS, Akt, PI3K, PDK1, and GLUT4 in MS rats with green tea and green coffee administration. The results showed that insulin signalling gene expressions were significantly higher in the intervention group compared to MS group rats. Moreover, the combination of green tea and green coffee had the highest expressions.

The present study aimed to investigate the effect of combining green tea and green coffee on cardiac insulin resistance caused by the elevation of FFA serum and lower adiponectin levels. This study showed that the administration of green tea and green coffee improved cardiac insulin resistance by reducing serum free fatty acid levels and elevating serum adiponectin levels by modulation of adipo-R1, AMPK, and FAS gene expressions. These results were similar to previous studies that showed improving serum FFA and adiponectin signalling pathways could attenuate insulin resistance. ^41^^–^^44^ Although animal and human studies have shown that lowering serum FFAs directly improved insulin resistance in some tissues such as muscles, adipose, hepatic, and endothelial tissues, few studies showed the effect on cardiac tissues. Moreover, green coffee administration could improve blood lipids and increase metabolic rates, fatty acid oxidation, and hepatic triglycerides in obese animal rat or mice models using diet or genetic modification. ^12^^,^^45^^–^^47^ In addition, a chlorogenic acid compound in the green coffee extract was associated with the improvement of adiponectin levels and had an important role in insulin sensitivity and inflammation. ^48^ Besides, green tea administration was known to have similar effects. ^49^ Recent study showed that the EGCG compound in green tea extract inhibited gene expression involved in synthesizing de novo fatty acids, such as FAS, ACC, and SC. ^50^ Other studies showed that EGCG had a beneficial effect on insulin resistance by improving the AMPK pathways. ^18^

In this study, the administration of a combination of green coffee and green tea extracts resulted in a higher adiponectin level compared to the administration of either green coffee or green tea extract alone. Additionally, the Adipo-R1 gene expression was higher in the CM group. Previous studies showed that green tea extract had a beneficial effect on the adiponectin signalling pathways by improving adiponectin levels and modulating adipo-R1 expression. ^37, 38^ On the other hand, few studies have revealed the effect of green coffee on the improvement of FFA levels. ^51^^,^^52^ Our study also indicated that green tea and green coffee administration were adequate to reduce serum FFA levels. This study was in accordance with previous studies that revealed the improvement of adiponectin and Adipo-R1 are involved in lower serum FFA levels. ^18^^,^^41^^,^^45^^–^^47^ The combination of both extracts exhibited lower serum FFA levels than green tea or decaffeinated light-roasted green coffee alone.

Elevation of serum FFA levels is caused by excess nutritional intake, adipose lipolysis, and de novo fatty acid synthesis (glucose utilization), mainly in the hepatic tissues. ^39^ The present study showed that hepatic de novo fatty acid synthesis was increased in metabolic syndrome rats. It was illustrated by increasing FAS mRNA expressions in the MS group (P < 0.01 vs. NC group). This finding was linear with our previous study that AMPK mRNA expressions were reduced in MS rats. ^34^ AMPK has an inhibitory effect on FAS ^53^; thus, the reduction of AMPK showed a decrease in inhibitory regulation effect. Nevertheless, green tea or green coffee intervention in the present study showed a decrease in FAS expressions, and these were similar to other previous studies ^45^^,^^46^^,^^51^^,^^54^^,^^55^; however, our findings showed no significant difference between green tea and green coffee extract rat groups. Meanwhile, the combination of light roasted decaffeinated green coffee and green tea extract significantly reduced FAS mRNA expression compared to that of single extract groups. These findings showed that green tea and green coffee had more practical effects on improving hepatic de novo fatty acid synthesis, thus reducing serum FFA levels.

It was known that FFA induced insulin resistance. ^44^^,^^56^ This study revealed HOMA-IR accompanied the increase of serum FFA levels in MS rats. Compared with previous studies, ^57^^–^^59^ we had similar findings that administration of green tea decaffeinated-light roasted green coffee had beneficial effects on improving insulin resistance. It was illustrated by a lowering of HOMA-IR in all intervention groups. Meanwhile, the administration of the combination of both extracts showed a lower level of HOMA-IR than green tea or green coffee administration alone. A previous study showed that the administration of EGCG ^54^^,^^60^ attenuated insulin sensitivity in skeletal and adipose tissue.. Moreover, CGA and other polyphenols in green coffee improved insulin sensitivity in HFD Mice. ^51^ HOMA-IR reflects insulin resistance conditions from multi organs such as adipose, heart, and muscle, including skeletal and cardiac muscle. ^61^^,^^62^

Many reviews have revealed that FFA could induce alteration of cardiac structure and function without coronary disease or hypertension, ^7^ which is called diabetic cardiomyopathy, and insulin resistance is the early step in the development of the disease. ^63^ Many studies suggest that an increase of serum FFA levels can induce insulin resistance by inhibiting IRS1/PI3K/Akt in cardiac muscle, ^64^^–^^66^ and associated with cardiac remodelling and dysfunction. ^67^^,^^68^ It was recently known that FFAs could interact with toll-like receptors (TLR) and induce insulin resistance through the disturbance of insulin signalling pathways in cardiac tissues. ^11^^,^^69^ Our findings observed that improvement of serum FFA levels was accompanied by improved cardiac insulin signalling pathways. All treatment groups showed a high of IRS1/2, PI3K, PDK1, Akt, and GLUT4 mRNA expression, but the GT group showed higher IRS2, Akt, and GLUT4 than the GC group. Rebollo-Hernanz *et al*. (2019) recently showed that phenolic compounds in green coffee, especially CGA, modulate adipogenesis and insulin resistance via PI3K/Akt signalling pathways in adipocytes. ^70^ Another study showed that chlorogenic acid stimulates glucose transport in skeletal muscle *via* AMPK activation in db/db mice. ^58^ Besides, the green tea administration showed the ameliorating mechanism of hyperglycemia by promoting GLUT4 translocation in skeletal muscle of diabetic rodents. ^60^ Moreover, another study revealed that EGCG administration induced GLUT4 translocation in skeletal muscle through PI3K- and AMPK-dependent pathways. ^16^ Nevertheless, the combination of green tea and decaffeinated-light roasted green coffee extract had the highest expressions. It showed that green tea and green coffee extract had synergistic interaction that consequently improved insulin sensitivity.

This study had several limitations. Firstly, we did not investigate the biomarker in adipose tissues, which might give a comprehensive understanding of green tea and green coffee administration’s effect on lipid metabolism. Secondly, we could not exhibit the molecular mechanisms for the effects of green tea and green coffee combination administration.

## Conclusion

Our study revealed clear evidence that a combination of green tea and decaffeinated light roasted green coffee extracts improved cardiac insulin resistance better than single extract administration by ameliorating free fatty acids through adiponectin/FAS pathways modulation.

## Data availability

Figshare: Underlying data for ‘Green Tea and decaffeinated light roasted green coffee extract combination improved cardiac insulin resistance through free fatty acids and adiponectin/FAS pathways amelioration in metabolic syndrome rat model’ https://doi.org/10.6084/m9.figshare.13249163.v3.

This project contains the following underlying data:

- Data of Metabolic Syndrome Rat Model.xlsx (This file contains the analyzed data)
- RAW data metabolic syndrome.xlsx (This file contains the actual observed values of the variables)
- Chlorogenic acid Coffee HPLC.pdf (This file contains the value of coffee chlorogenic acid levels as measured by HPLC)
- Caffeine coffee HPLC.pdf (This file contains the value of coffee caffeine levels as measured by HPLC)
- Green Tea Catechin HPLC.pdf (This file contains the value of the catechin levels in the tea)

Data are available under the terms of the Creative Commons Attribution 4.0 International license (CC-BY 4.0).

## Author contributions

M.L: Conceptualization, Data Curation, Methodology, Writing – Review & Editing

M.S.R: Conceptualization, Funding acquisition, Supervision, Writing – Review & Editing

D.A.N: Conceptualization, Data Curation, Methodology, Resources

M.N.R: Data Curation, Formal Analysis, Investigation, Writing – Original Draft Preparation

N.A.W: Data Curation, Investigation, Writing – Original Draft Preparation

N.W: Project Administration, Supervision, Writing – Review & Editing
