# Supplementary material for: Green Tea and Decaffeinated Light Roasted Green Coffee Extract Combination Improved Cardiac Insulin Resistance through Free Fatty Acids and Adiponectin/FAS Pathways Amelioration in Metabolic Syndrome Rat Model
Source: F1000Res. 2026 Jul 3;10:990. Originally published 2021 Sep 30. [Version 3] doi: 10.12688/f1000research.55470.3 (PMC13554843; doi:10.12688/f1000research.55470.3)
Supplement: Peer review history

# Reviewer report: Reviewer response for version 3

**DOI:** 10.5256/f1000research.203394.r500479

**Published:** 2026-08-07

**Authors:** Andi Ariyandy

**Affiliations:** 1 Department of Physiology, Faculty of Medicine, Hasanuddin University, Makassar, South Sulawesi, Indonesia

This manuscript investigates whether a combination of green tea extract and decaffeinated light-roasted green coffee extract improves metabolic abnormalities and cardiac insulin-signaling-related gene expression in a rat model of metabolic syndrome. Twenty-five male Sprague–Dawley rats were divided into five groups: normal control, metabolic syndrome, green tea, green coffee, and combination treatment. Metabolic syndrome was induced using a high-fat, high-sucrose diet and low-dose streptozotocin. After nine weeks of treatment, the authors measured serum non-esterified fatty acids, adiponectin, fasting glucose and insulin-derived HOMA-IR, hepatic Adipo-R1 and FAS expression, and cardiac IRS1/2, PI3K, PDK1, Akt, and GLUT4 mRNA expression. The combination group generally showed more favorable results than the untreated metabolic syndrome group and, for several outcomes, than the single-extract groups. The topic is relevant, and the overall biological direction of the findings is plausible. However, substantial methodological, statistical, and interpretative concerns must be addressed before the manuscript can be considered scientifically sound.

 Clarity and current literature: Partly

 The research question and general study structure are understandable, and the manuscript cites literature that supports the relationships among free fatty acids, adiponectin, insulin resistance, and cardiac insulin signaling. Nevertheless, the presentation contains multiple inconsistencies in terminology, assay descriptions, measurement units, and interpretation.

 For example, serum and plasma are used interchangeably, the reported NEFA measurement method and units are inconsistent, and the HPLC results mention three analytes but provide only two concentrations. There are also clear textual errors, such as the statement that the green tea group had lower adiponectin than the green tea group. These errors make interpretation difficult and require comprehensive scientific and language editing.

 In addition, the reference list appears largely limited to studies published up to 2021. For a manuscript revised in 2026, the Introduction and Discussion should be updated to reflect recent literature on cardiac insulin resistance, adiponectin signaling, chlorogenic acid and catechins, tissue-specific insulin action, the standardization of herbal extracts, and current standards for animal research.

 Study design and technical soundness: Partly

 The five-group design is broadly appropriate for comparing untreated metabolic syndrome, single-extract treatments, and combined treatment. However, the manuscript does not clearly report randomization, blinding, sample size justification, exact allocation per group, attrition, exclusion criteria, or whether control animals received an equivalent vehicle gavage.

 Although 25 animals were enrolled, the figure legends report sample sizes of four to five animals per group. The authors must explain all missing observations, failed assays, exclusions, deaths, or unavailable tissue samples. The experimental unit should also be defined, particularly because animals may have been housed in shared cages.

 The streptozotocin protocol is insufficiently described. The authors should report the exact dose, solvent, injection volume, number and timing of injections, fasting conditions, and criteria used to confirm successful induction. The operational definition of metabolic syndrome in rats should also be provided, rather than relying solely on the human NCEP ATP III terminology.

 Methods sufficient for replication: No

 Important procedural information remains incomplete. The authors must provide complete green tea and green coffee extraction procedures, extraction yields, decaffeination method, residual caffeine content, extract concentrations, gavage volume, vehicle, storage conditions, and stability. Full HPLC validation and the amounts of EGCG, chlorogenic acid, caffeine, and total polyphenols administered to each animal should be reported clearly.

 The gene-expression method is particularly unclear. The manuscript describes quantitative PCR using a LightCycler and a touchdown PCR protocol. Still, the reported PCR reagent and methodological description do not clearly establish whether real-time quantitative PCR or conventional endpoint PCR was performed.

 The authors must clarify the exact method and report the reagent, fluorescent detection chemistry where applicable, cycling conditions, amplification efficiency, amplicon size, specificity testing, technical replicates, negative controls, RNA integrity, and normalization procedure. Raw Ct values or original uncropped gel images should be made available. The stability of β-actin as the reference gene across the experimental groups should also be demonstrated.

 Statistical analysis and interpretation: No

 The authors used multiple independent pairwise *t*-tests across five groups without an omnibus test or correction for multiple comparisons. This approach substantially increases the risk of false-positive findings.

 The four metabolic-syndrome groups represent a natural 2 × 2 factorial design based on the presence or absence of green tea and green coffee. A factorial ANOVA or equivalent general linear model should be used to estimate the main effect of each extract and the green tea × green coffee interaction. The interaction term is essential because the manuscript repeatedly claims that the treatments have a synergistic effect. Demonstrating that the combination group differs from either monotherapy group does not, by itself, establish synergy.

 The revised analysis should include assumption testing, multiplicity-adjusted comparisons, exact *p* values, effect sizes, 95% confidence intervals, and exact sample sizes. Individual-animal data should be displayed rather than bar graphs alone.

 The HOMA-IR calculation also requires urgent verification. The authors should justify the selected formula, denominator, glucose units, insulin units, fasting duration, and applicability to Sprague–Dawley rats. If the calculation is incorrect, all values and associated analyses must be repeated. HOMA-IR should also be described as a surrogate measure of systemic fasting insulin resistance, rather than a direct measure of cardiac insulin resistance.

 Source data and reproducibility: Partly

 Providing a Figshare repository containing analyzed data, raw spreadsheets, and HPLC files is a strength. However, it is unclear whether the repository contains individual-animal identifiers, group allocation, attrition records, raw absorbance values, standard curves, assay dilution factors, PCR source data, missing-data indicators, and complete statistical syntax.

 The authors should add a data dictionary and clearly map each source-data variable to every figure panel and statistical analysis.

 Conclusions supported by results: Partly

 The results support associations between combined extract administration, lower serum NEFA, improved systemic insulin-resistance indices, and altered hepatic and cardiac insulin-signaling-related mRNA expression. They do not directly demonstrate myocardial insulin resistance, improved cardiac function, protein activation, GLUT4 translocation, pharmacological synergy, or a causal adiponectin/FAS mechanism.

 The title, abstract, Discussion, and Conclusion should therefore use cautious wording such as “associated with,” “accompanied by,” or “may involve.” Terms such as “synergistic,” “mediated through,” and “clear evidence” should be removed unless supported by appropriate interaction analyses or additional mechanistic experiments.

 Overall recommendation

**Not approved in its current form.** The research question is potentially valuable, but major revision and statistical reanalysis are required. The statistical method, HOMA-IR calculation, assay reporting, PCR methodology, animal-study details, extract characterization, source-data completeness, and overstated mechanistic conclusions must be addressed before the reliability and scientific significance of the findings can be adequately evaluated.

Is the work clearly and accurately presented and does it cite the current literature?

Partly

If applicable, is the statistical analysis and its interpretation appropriate?

No

Are all the source data underlying the results available to ensure full reproducibility?

Partly

Is the study design appropriate and is the work technically sound?

Partly

Are the conclusions drawn adequately supported by the results?

Partly

Are sufficient details of methods and analysis provided to allow replication by others?

No

Reviewer Expertise:

Endocrine Cardiometabolic Physiology

I confirm that I have read this submission and believe that I have an appropriate level of expertise to state that I do not consider it to be of an acceptable scientific standard, for reasons outlined above.

# Reviewer report: Reviewer response for version 3

**DOI:** 10.5256/f1000research.203394.r500483

**Published:** 2026-07-28

**Authors:** Tran Chi Linh

**Affiliations:** 1 Faculty of Medicine, College of Health Sciences,, Nam Can Tho University, Can Tho City, Vietnam

The revised manuscript has been substantially improved and is now much stronger than the previous version. The authors have addressed many of the major concerns raised during the earlier review, resulting in a clearer and more comprehensive presentation of the study.

 However, several issues still require attention before the manuscript can be considered for indexing.

 First, the conclusions should be more closely aligned with the actual findings of the study. The authors are encouraged to avoid overly broad or generalized statements that extend beyond the evidence presented. Instead, the conclusions should accurately reflect the experimental results and acknowledge the scope and limitations of the work.

 In addition, the manuscript would benefit from a thorough review of its English grammar, sentence structure, and overall language quality to improve clarity and readability.

 Finally, the quality and resolution of several figures should be enhanced to ensure that all details are clearly visible and meet the journal's standards.

Is the work clearly and accurately presented and does it cite the current literature?

Yes

If applicable, is the statistical analysis and its interpretation appropriate?

Yes

Are all the source data underlying the results available to ensure full reproducibility?

Yes

Is the study design appropriate and is the work technically sound?

Yes

Are the conclusions drawn adequately supported by the results?

Yes

Are sufficient details of methods and analysis provided to allow replication by others?

Yes

Reviewer Expertise:

Drug Discovery from Natural Sources

I confirm that I have read this submission and believe that I have an appropriate level of expertise to confirm that it is of an acceptable scientific standard.

# Reviewer report: Reviewer response for version 3

**DOI:** 10.5256/f1000research.203394.r500474

**Published:** 2026-07-15

**Authors:** Igbayilola Yusuff Dimeji

**Affiliations:** 1 Department of Physiology, College of Medicine, Federal University of Health Sciences, Ila-Orangun, Osun State, Nigeria

A major strength of this study is its investigation of the synergistic therapeutic effects of combining green tea extract and decaffeinated light-roasted green coffee extract, rather than evaluating either intervention alone, thereby providing a more clinically relevant nutraceutical strategy for the management of metabolic syndrome. The work is further distinguished by its elucidation of the mechanistic basis of cardioprotection, demonstrating improvements in cardiac insulin resistance through modulation of free fatty acid metabolism and the adiponectin/FAS signaling pathway. By integrating biochemical, molecular, and metabolic assessments in a well-established rat model of metabolic syndrome, the study provides robust evidence linking metabolic regulation to improved cardiac function. These findings enhance the translational potential of plant-derived bioactive combinations as complementary therapeutic interventions for preventing cardiovascular complications associated with metabolic syndrome.

 However, I have the following concerns that should be addressed before the manuscript can be considered for indexing:

1. **Abstract:** The Methods section of the abstract lacks a brief description of the statistical analyses performed, while the Results section does not report the level of statistical significance (e.g., *p* < 0.05). Please revise the abstract to include these details to improve its scientific rigor and logical flow.
2. **Keywords:** Revise the keywords to avoid repeating terms already included in the title. Select complementary keywords that improve the manuscript's discoverability.
3. **Introduction:** The epidemiological burden of metabolic syndrome is inadequately described. Please include one or two sentences highlighting its global and regional prevalence, with emphasis on geographical distribution, to strengthen the rationale for the study.
4. **Methods:** The sections describing animal grouping, metabolic syndrome induction, anaesthesia, and blood and tissue sample collection are presented together. These should be reorganized into separate subsections to improve readability and methodological clarity.
5. **Control Group:** The authors stated that the normal control (NC) group received normal chow without any vehicle administration. This constitutes a methodological concern because control animals should receive the same vehicle used for administering the experimental treatments (e.g., distilled water or normal saline). Please justify this omission or clarify whether a vehicle was administered.
6. **Methodological Organization:** The description of the extraction procedures should precede the sections on animal grouping, induction of metabolic syndrome, anaesthesia, and sample collection to improve the logical progression of the methodology.
7. **Assessment of Glucose Homeostasis:** Since this is a metabolic syndrome study, the inclusion of oral glucose tolerance test (OGTT) and insulin tolerance test (ITT) data would provide valuable evidence of glucose tolerance and insulin sensitivity. If these data are available, they should be included in the revised manuscript.
8. **Pancreatic Assessment:** The study would be significantly strengthened by evaluating pancreatic β-cell function and pancreatic histopathology to determine whether the treatments promote structural and functional recovery of pancreatic tissue.
9. **Positive Control:** A standard antidiabetic drug, such as metformin, should have been included as a positive control to enable comparison of the therapeutic efficacy of the plant extracts with an established pharmacological intervention.
10. **Adiponectin Measurement and Physiological Parameters:** Please specify the tissue or biological sample in which adiponectin was measured. In addition, data on weekly food intake and body weight changes should be presented, as these are important indicators in metabolic syndrome studies.
11. **Correlation and Cardiovascular Data:** Were correlation analyses performed between food intake and body weight changes? Furthermore, blood pressure measurements were described in the Methods section, yet no corresponding results are presented. Please clarify this discrepancy or include the relevant data.
12. **Additional Metabolic Markers:** The absence of GLUT-4 expression analysis and lipid profile assessment represents a major limitation, given that these are key biomarkers in metabolic syndrome research. Their inclusion would substantially strengthen the mechanistic interpretation of the findings.
13. **Discussion:** The Discussion should be expanded by integrating the present findings with previous studies and providing a more comprehensive explanation of the underlying mechanisms responsible for the observed cardiometabolic effects.
14. **Overall Presentation:** The manuscript would benefit from substantial organisational restructuring and thorough language editing to improve clarity, coherence, and overall readability.

Is the work clearly and accurately presented and does it cite the current literature?

Partly

If applicable, is the statistical analysis and its interpretation appropriate?

Yes

Are all the source data underlying the results available to ensure full reproducibility?

No source data required

Is the study design appropriate and is the work technically sound?

Partly

Are the conclusions drawn adequately supported by the results?

Partly

Are sufficient details of methods and analysis provided to allow replication by others?

No

Reviewer Expertise:

Nutrition, Endocrinology and Meatbolism

I confirm that I have read this submission and believe that I have an appropriate level of expertise to confirm that it is of an acceptable scientific standard, however I have significant reservations, as outlined above.

# Reviewer report: Reviewer response for version 3

**DOI:** 10.5256/f1000research.203394.r498220

**Published:** 2026-07-08

**Authors:** Vijaya Anand Arumugam

**Affiliations:** 1 Department of Human Genetics and Molecular Biology, Bharathiar University, Coimbatore, Tamil Nadu, India

**Comments to the editor**

 The authors have put effort to evaluate the “Green Tea and Decaffeinated Light Roasted Green Coffee Extract Combination Improved Cardiac Insulin Resistance through Free Fatty Acids and Adiponectin/FAS Pathways Amelioration in Metabolic Syndrome Rat Model”. The authors have carried out the necessary corrections suggested by me. I have checked only the topics of my remarks and in general in the revised manuscript and it has been noted few shortcomings. Hence, the paper can be ACCEPTED after incorporating the correction with MINOR REVISIONS and it does not require re-review.

**Comments to the authors**

 1. This suggestion is not carried out properly and it should be rectified (Check the abbreviations throughout the manuscript and introduce the abbreviation when the full word appears first time in the abstract and the remaining for the text and then use only the abbreviation). For example, metabolic syndrome (MS), chlorogenic acid (CGA), HFD, control group (NC), a metabolic syndrome group (MS). and these types of corrections need to be checked all other abbreviations used in the manuscript.

Is the work clearly and accurately presented and does it cite the current literature?

Yes

If applicable, is the statistical analysis and its interpretation appropriate?

Yes

Are all the source data underlying the results available to ensure full reproducibility?

Yes

Is the study design appropriate and is the work technically sound?

Yes

Are the conclusions drawn adequately supported by the results?

Yes

Are sufficient details of methods and analysis provided to allow replication by others?

Yes

Reviewer Expertise:

Phytotherapeutics and Clinical Biochemistry

I confirm that I have read this submission and believe that I have an appropriate level of expertise to confirm that it is of an acceptable scientific standard, however I have significant reservations, as outlined above.

# Reviewer report: Reviewer response for version 2

**DOI:** 10.5256/f1000research.155894.r444953

**Published:** 2026-01-07

**Authors:** Doha Reda

**Affiliations:** 1 Clinical pathology, Mansoura University Faculty of Veterinary Medicine (Ringgold ID: 158400), Mansoura, Dakahlia Governorate, Egypt

**General Impression**

 The authors investigated whether a combination of green tea and decaffeinated light-roasted green coffee extract has a superior effect on improving cardiac insulin resistance compared with single treatments. It presents an interesting research concept with a specific focus on cardiac tissue, clear figures, and a well-structured manuscript. However, it also has several weaknesses, which are outlined below:

**Abstract **

- The control group should be mentioned first, followed by the metabolic syndrome group, to maintain consistency throughout the manuscript.
- This study uses a relatively small sample size.
- The abbreviations HFHS and STZ should be defined.
- The statistical significance is not included.

**Introduction**

       There are some grammatical errors, such as: “A pervious study were” in the 20 ^th^ line should be “A pervious study was”; “by improving the adiponectin/FAS pathways” should be “improving the adiponectin/FAS pathway.”

**Method**

- The number of animals per group should be specified.
- The composition of both the normal and high-fat, high-sucrose diets should be detailed.
- The dose of streptozotocin and the frequency of administration per week should be clearly stated.
- In line 9, “administration per oral” should be corrected to “administered orally.”
- The description of the Homeostatic Model Assessment for Insulin Resistance (HOMA-IR) is unclear. The authors mention that it was used to calculate an index, but they do not explain what this index represents.
- For clarity, the primer sequences in the PCR protocol should be presented in a table.
- One-way ANOVA, rather than independent t-tests, should be applied to evaluate statistical differences among all tested groups, since t-tests can only compare two groups at a time.

**Results**

- Data on food intake and body weight are missing.
- The manuscript lacks reference to the NCEP-ATP III criteria, which should be cited and justified for defining metabolic syndrome.
- The authors should clarify whether free fatty acids were measured in the plasma or in the serum.
- The figure numbering does not align with the text; specifically, the legend of Figure 2 should be replaced with Figure 3, and vice versa.

**Discussion**

 This sentence should be added: “Future studies are needed to translate these findings to humans.”

Is the work clearly and accurately presented and does it cite the current literature?

Partly

If applicable, is the statistical analysis and its interpretation appropriate?

No

Are all the source data underlying the results available to ensure full reproducibility?

Partly

Is the study design appropriate and is the work technically sound?

Partly

Are the conclusions drawn adequately supported by the results?

Partly

Are sufficient details of methods and analysis provided to allow replication by others?

No

Reviewer Expertise:

Metabolic diseases, Nutrition, Clinical chemistry

I confirm that I have read this submission and believe that I have an appropriate level of expertise to state that I do not consider it to be of an acceptable scientific standard, for reasons outlined above.

# Reviewer report: Reviewer response for version 1

**DOI:** 10.5256/f1000research.59048.r138844

**Published:** 2022-07-18

**Authors:** Sylvia Riedel

**Affiliations:** 1 Biomedical Research and Innovation Platform, South African Medical Research Council (MRC), Tygerberg, South Africa

**General impression:** The authors determined whether green tea, decaffeinated green coffee, and a combination thereof can reduce features of metabolic syndrome in rats, with a specific focus on insulin resistance. The manuscript in its current form lacks important information as well as clarity. Grammar and sentence construction need to be reviewed critically throughout the manuscript. The work has merit, but there are too many errors and omissions in this version, the authors should rethink and revise the manuscript thoroughly. The referencing requires attention. Specific comments are highlighted below:

**Abstract**

- Second sentence - grammar. What does “improvement of free fatty acid” mean?
- Abbreviations need to be explained when first used (e.g. FAS).
- It is unclear how many rats were used per group, is it 25 for all groups or per group?
- From the abstract, it is not clear whether groups 3, 4, and 5 were given the high-fat high-sucrose (HFHS) diet and streptozotocin (STZ).
- What is the rationale for the combination group of green tea and green coffee?
- Was circulating adiponectin measured and in which tissues were the mentioned genes assessed?
- The results should be stated clearly and vague statements such as “showed synergistic effects” should be avoided.
- The p values should be included. A clear description of which parameters increased and which decreased in which groups should be included.

**Introduction**

- There is a specific set of diagnostic criteria for metabolic syndrome which should be detailed and referenced in the introduction. Reference 10 describes non-alcoholic fatty liver disease (NAFLD) and not insulin resistance (IR) as such, it seems inappropriate to cite here. There are a few sentences that do not make sense. The authors should draw a distinction between cardiac dysfunction and cardiac insulin resistance and define what cardiac insulin resistance entails.

**Methods**

- The study outline is not clear enough from the description, an illustration would be helpful. Critical details regarding the animal work are missing, such as the number of animals per group and dosage of STZ applied. What are the “NCEP ATP III” criteria? These need to be explained and referenced and how the rats used for this study meet these criteria needs to be presented with data (can be supplementary data).
- What is the composition of the HFHS diet?
- It seems that this study and the study by Rohman *et al.* 2021 (reference 31) are using the same animals. If this is the case, then this should be stated clearly. Both manuscripts could benefit from combining the results to provide a more substantial and comprehensive overview of the study in one single paper.
- It would be preferable if critical references such as 31 were already published/accepted articles, rather than cited while awaiting peer review.   
- If the present study and reference 31 are different experiments, then critical data on the animal’s metabolic status (fasting blood glucose, body weights, and data showing that metabolic syndrome was induced, etc.) needs to be provided in the manuscript (the data is shared, but clear visuals would be preferable) and all paragraphs in the methods including extract preparation and the animal study need to clearly reflect this.
- There is also mention that the study is part of a study published previously (reference 30) – it needs to be clarified in which way. It seems that some of the confusion derives from an unclear formulation of sentences.
- The preparation of the extracts can be referenced if the same method was followed as in reference 31, however, it is not clear what was used to gavage the animals and how the solutions were prepared. Were the animals in the normal control group and the metabolic syndrome control also gavaged and if so, with what? For future studies, alternative methods such as administering the extracts in the diet should be considered to replace gavaging. Extracts can be freeze-dried and mixed into the diet.
- It is not clear how the Homeostatic Model Assessment for Insulin Resistance (HOMA-IR) was analyzed using a kit. How was fasting blood glucose determined? This section requires clarification.
- Statistical analysis using a t-test is inappropriate. To assess group differences at least a one-way analysis of variance (ANOVA) or better yet a two-way ANOVA should be used for the MS-induced treatment groups. The MS treatment groups fit a 2x2 factorial design, and a two-way ANOVA will give more appropriate insight into the data, especially the combination of treatments. The negative control and positive control can be compared using a t-test. It is unclear whether the authors report standard deviations (SD) as stated in the statistics paragraph or standard error of the mean (SEM) as stated in the graph legends. The distinction is important and requires clarification.

**Results**

- Legends to the graphs require more details, such as an explanation of abbreviations and a clear description in which tissues gene expression was analyzed. In general, graph legends should provide enough information to understand the data presented without reading the text.
- It was stated that decaffeinated green coffee was used. An explanation is required as to why the extract seems to contain caffeine. There is a chromatogram for caffeine in the data section. The second sentence in the HPLC results section is not clear, it mentions CGA, caffeine, and polyphenols but only two values are given. These values should also be presented with standard deviations of replicate measurements. This needs clarification.
- Graph numbers do not align with the references in the text. For example, on page 6 the first paragraph refers to Fig 2, but the data is displayed in Fig 3. In the last paragraph on page 10, the authors also refer to Fig 2 which should be Fig 3.
- There was no protein expression included or reported. This is an important aspect to add as gene expression does not necessarily correlate with protein levels and function.

**Discussion**

- Once the manuscript structure has been clarified and revised, the discussion should be refocused and written concisely to clearly work out what new knowledge this study brings to the body of literature. Grammar should be checked.
- The last sentence in the limitations section “…we could not exhibit the molecular mechanisms for the effects of green tea and green coffee combination administration…”, is unclear but seems to contradict the authors' conclusion.

Is the work clearly and accurately presented and does it cite the current literature?

No

If applicable, is the statistical analysis and its interpretation appropriate?

No

Are all the source data underlying the results available to ensure full reproducibility?

Partly

Is the study design appropriate and is the work technically sound?

Partly

Are the conclusions drawn adequately supported by the results?

Partly

Are sufficient details of methods and analysis provided to allow replication by others?

Partly

Reviewer Expertise:

Metabolic diseases, inflammation, gastrointestinal physiology, food chemistry

I confirm that I have read this submission and believe that I have an appropriate level of expertise to state that I do not consider it to be of an acceptable scientific standard, for reasons outlined above.

# Reviewer report: Reviewer response for version 1

**DOI:** 10.5256/f1000research.59048.r98201

**Published:** 2021-11-10

**Authors:** Vijaya Anand Arumugam

**Affiliations:** 1 Department of Human Genetics and Molecular Biology, Bharathiar University, Coimbatore, Tamil Nadu, India

The authors intended to evaluate the effects of Green Tea and Decaffeinated Light Roasted Green Coffee Extract metabolic syndrome. It is an interesting and useful study. Firstly, this manuscript is well-organised and well-structured. Secondly, this study observed the differentially expressed genes and provided potential target candidates for the treatment of the metabolic syndrome.

 1. There are some grammatical and syntax errors in the manuscript. For example, the words “A free fatty acid is” may be as “Free fatty acids are”; “improvement” as “the improvement” (in the 7th line of the abstract); “IRS-1” as “of IRS-1”; “Combination green” as “A combination of green”; “study also” as “study were also”; “higher of” as “higher”; “lower of” as “lower”; “different with” as “different from”. The grammar mistakes which are not mentioned here also to be checked and corrected properly.

 2. There are some typing mistakes as well, and authors are advised to carefully proof-read the text. For example, the word “prodcedure” may be as “procedure”; “compound level” as “compound levels”; “tothe” as “to the”; “extract treated” as “extract-treated”; “theMSgroup” as “the MS group”; “theNCgroup” as “the NC group”; “GTgroup” as “GT group”; “theGTandGCgroups” as “the GT and GC groups”; “theCMgroup” as “the CM group”; “injection induced” as “injection-induced”; “pathway” as “pathways”; “tissue..” as “tissue.”; “decaffeinatedlight” as “decaffeinated light”. The typos not mentioned here also to be checked and corrected properly.

 3. The keywords should not be from the title and also the remaining part of the manuscript. The keywords should assist computer searches to find your specific article.

 4. Check the abbreviations throughout the manuscript and introduce the abbreviation when the full word appears the first time in the text and then use only the abbreviation (For example, high-fat high sucrose (HFHS), streptozotocin (STZ), metabolic syndrome (MS), chlorogenic acid (CGA), high-fat diet (HFD), etc.). And it should be in both abstract as well as in the remaining part of the manuscript. Make a term abbreviated in the article when it is repeated at least three times in the text, not all of them need to be.

 6. The weight of the animals should be included in the materials and methods under the heading “Animals and experimental design”. The composition of the standard diet may be included in the materials and methods and also where it has been obtained.

 7. In the statistical analysis the authors should include the software and version of the software used in the present findings under the heading “Data and statistical analysis”.

 8. The referencing in this paper is inconsistent. The authors have not given the references as per the journal format. In many places, the authors used numbers and authors with year and in few places used only numbers and in some places the authors' used names and years only. For example, “Van Dijk *et al*., 2013” and it should be corrected properly.

 9. The figures should be improved and a proper footnote should be given. All legends should have enough description for a reader to understand the figures without having to refer back of the main text of the manuscript. The authors have used the abbreviations in the axis and expansions that are not given with the graph. The quality of the image is not to be changed since it seems good. The only suggestion here is the authors may include the expansion of the terms used like 1) MS (metabolic syndrome), 2) NC (normal chow) 3) GT (green tea extract) 4) GC (decaffeinated-light roasted green coffee extract), 5) CM (combination of both extracts) as legends.

 10. The author should justify how the dose fixation is done (is it based on toxicity test or previous reference, if previous should be included in the reference section) in materials and methods or discussion section of the manuscript.

Is the work clearly and accurately presented and does it cite the current literature?

Yes

If applicable, is the statistical analysis and its interpretation appropriate?

Yes

Are all the source data underlying the results available to ensure full reproducibility?

Yes

Is the study design appropriate and is the work technically sound?

Yes

Are the conclusions drawn adequately supported by the results?

Yes

Are sufficient details of methods and analysis provided to allow replication by others?

Yes

Reviewer Expertise:

Phytothreapeutics, Clinical Biochemistry, Medical Genetics

I confirm that I have read this submission and believe that I have an appropriate level of expertise to confirm that it is of an acceptable scientific standard, however I have significant reservations, as outlined above.

# Response

**Published:** 2023-09-10

**Authors:** Dwi Adi Nugroho

**Affiliations:** Brawijaya University, Indonesia

Dear A. Vijaya Anand, 

 Department of Human Genetics and Molecular Biology, Bharathiar University, Coimbatore, Tamil Nadu, India

 I would like to express my sincere gratitude for taking the time to review our manuscript titled "Green Tea and Decaffeinated Light Roasted Green Coffee Extract Combination Improved Cardiac Insulin Resistance through Free Fatty Acids and Adiponectin/FAS Pathway Amelioration in Metabolic Syndrome Rat Model." Your thoughtful feedback and constructive comments have been invaluable in improving the quality of our work.

 I have made the necessary revisions to the manuscript as per your recommendations. You will find these changes clearly marked in the revised manuscript. Additionally, I have included a detailed response to each comment in the attached document.

 Once again, thank you for your time and expertise in reviewing our work. We believe that your feedback has significantly enhanced the quality and rigor of our research. We look forward to your further insights and guidance during the next round of review.

 Sincerely,

 M.N. Kholis
